# Sex and parasites: genomic and transcriptomic analysis of *Microbotryum lychnidis-dioicae,* the biotrophic and plant-castrating anther smut fungus

**DOI:** 10.1186/s12864-015-1660-8

**Published:** 2015-06-16

**Authors:** Michael H Perlin, Joelle Amselem, Eric Fontanillas, Su San Toh, Zehua Chen, Jonathan Goldberg, Sebastien Duplessis, Bernard Henrissat, Sarah Young, Qiandong Zeng, Gabriela Aguileta, Elsa Petit, Helene Badouin, Jared Andrews, Dominique Razeeq, Toni Gabaldón, Hadi Quesneville, Tatiana Giraud, Michael E. Hood, David J. Schultz, Christina A. Cuomo

**Affiliations:** Department of Biology, Program on Disease Evolution, University of Louisville, Louisville, KY 40292 USA; Institut National de la Recherche Agronomique (INRA), Unité de Recherche Génomique Info (URGI), Versailles, France; Institut National de la Recherche Agronomique (INRA), Biologie et gestion des risques en agriculture (BIOGER), Thiverval-Grignon, France; Ecologie, Systématique et Evolution, Bâtiment 360, Université Paris-Sud, F-91405 Orsay, France; CNRS, F-91405 Orsay, France; Broad Institute of MIT and Harvard, Cambridge, MA 02142 USA; INRA, UMR 1136, Interactions Arbres-Microorganismes, Champenoux, France; UMR 1136, Université de Lorraine, Interactions Arbres-Microorganismes, Vandoeuvre-lès-Nancy, France; Centre National de la Recherche Scientifique (CNRS), UMR7257, Université Aix-Marseille, 13288 Marseille, France; Department of Biological Sciences, King Abdulaziz University, Jeddah, Saudi Arabia; Centre for Genomic Regulation (CRG), Barcelona, Spain; Universitat Pompeu Fabra (UPF), Barcelona, Spain; Institució Catalana d’Estudis Avançats (ICREA), Barcelona, Spain; Department of Biology, Amherst College, Amherst, MA 01002 USA

**Keywords:** *Microbotryum violaceum*, Anther smuts, CAZymes, Transposable elements, Mating-type chromosomes, Pathogen alteration of host development

## Abstract

**Background:**

The genus *Microbotryum* includes plant pathogenic fungi afflicting a wide variety of hosts with anther smut disease. *Microbotryum lychnidis-dioicae* infects *Silene latifolia* and replaces host pollen with fungal spores, exhibiting biotrophy and necrosis associated with altering plant development.

**Results:**

We determined the haploid genome sequence for *M. lychnidis-dioicae* and analyzed whole transcriptome data from plant infections and other stages of the fungal lifecycle, revealing the inventory and expression level of genes that facilitate pathogenic growth. Compared to related fungi, an expanded number of major facilitator superfamily transporters and secretory lipases were detected; lipase gene expression was found to be altered by exposure to lipid compounds, which signaled a switch to dikaryotic, pathogenic growth. In addition, while enzymes to digest cellulose, xylan, xyloglucan, and highly substituted forms of pectin were absent, along with depletion of peroxidases and superoxide dismutases that protect the fungus from oxidative stress, the repertoire of glycosyltransferases and of enzymes that could manipulate host development has expanded. A total of 14 % of the genome was categorized as repetitive sequences. Transposable elements have accumulated in mating-type chromosomal regions and were also associated across the genome with gene clusters of small secreted proteins, which may mediate host interactions.

**Conclusions:**

The unique absence of enzyme classes for plant cell wall degradation and maintenance of enzymes that break down components of pollen tubes and flowers provides a striking example of biotrophic host adaptation.

**Electronic supplementary material:**

The online version of this article (doi:10.1186/s12864-015-1660-8) contains supplementary material, which is available to authorized users.

## Background

Members of the genus *Microbotryum* are pathogenic fungi that have a global distribution and infect over nine families of host plants, with most replacing floral structures with fungal spores [1]. Within this genus, the anther-smut fungi of plants in the Caryophyllaceae, consist of many recognized and cryptic species, most possessing a very narrow host range, although some are more generalist [2]. Hybrid incompatibilities for the species examined in this complex leads to post-zygotic isolation in the forms of inviability and sterility [3–5]. The high rates of selfing and ecological specialization on different host plants are factors that should promote speciation in *Microbotryum* [6, 7]. The anther smut fungi thus allow examination of the ecology and evolution of host/pathogen interactions in “wild,” non-agricultural environments [8, 9], where the genetically variable hosts provides an important contrast to the heavily studied, more monocultural hosts of agricultural systems. *Microbotryum* species also serve as a model for emerging infectious disease through host shifts [9, 10], for studying the evolution of mating systems, non-recombining mating-type chromosomes and sex chromosomes [11, 12], and for examining pathogens that alter the development of the host [13].

**Fig. 1:**
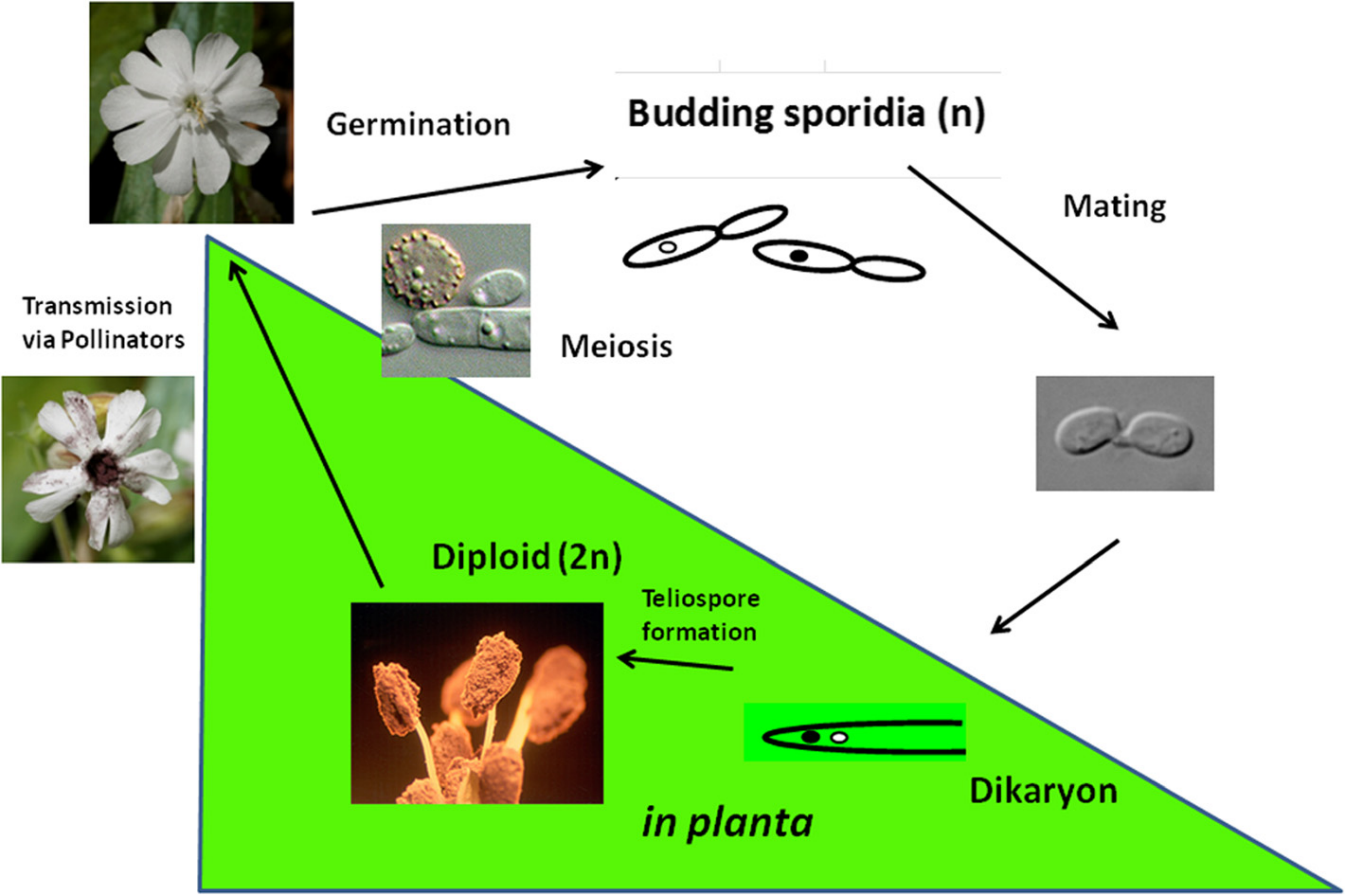
Lifecycle of *Microbotryum lychnidis-dioicae*. The infection cycle for *M. lychnidis-dioicae*) is shown. Infection begins when diploid teliospores germinate on a suitable plant surface, and after meiosis, produce linear tetrads of haploid basidiospores. These cells may mate with cells of opposite mating-type (*i.e.*, *a1* or *a2*), either directly within the tetrad or after budding to yield free yeast-like sporidia. Mated cells form conjugation bridges and, upon receiving suitable cues from the host plant, develop into dikaryotic hyphae that penetrate the host tissue. The hyphae progress systemically through the plant and migrate to the flower primordia. There nuclear fusion (karyogamy) occurs, as the hyphae break up and develop into diploid teliospores that replace the pollen in the anthers of the developing flowers. Pollinator species are then able to transmit the spores to other uninfected flowers, thus completing the cycle

As obligate parasites, *Microbotryum* anther smut fungi must complete their life cycle in association with a host plant. Their fungal diploid teliospore masses give the flowers a dark, powdery appearance, thus the name “anther smut.” Teliospores of *Microbotryum* are transported from diseased to healthy plants by direct transmission when plants are in close proximity [14] or by pollinating insects [15], where once deposited the diploid fungus germinates and undergoes meiosis to give rise to four haploid cells [6]. Each of these cells can bud off yeast-like sporidia on the plant surface. New infectious dikaryotic hyphae are rapidly produced by conjugation of two cells of opposite mating-type (*a*_*1*_ and *a*_*2*_) and enter the host tissue to grow endophytically until they reach the bud meristems and anthers [16]. Here the nuclei fuse (karyogamy) and teliosporogenesis occurs thus completing the life cycle (Fig. 1; [6, 17–19]).

The commandeering of insect pollinators for disease transmission is associated with significant pathogenic alterations of the host’s floral morphology, particularly in relation to male and female structures. Although diseased plants are only slightly affected in vegetative morphology and survival [20], infection often results in complete host sterility, as no pollen is produced in the anthers and the ovary becomes rudimentary. Interestingly, in dioecious or gynodioecious species of *Silene* (*e.g.*, *S. latifolia* and *S. vulgaris*, respectively), ovaries of diseased female plants are aborted while a male morphology develops with spore-bearing anthers. The basis for the changes in female plants is not fully understood. Studies have identified host genes expressed during infection of females that are also normally expressed in uninfected males (*MEN* genes [21]; *SLM2* [22]).

The formation of a hyphal dikaryon by mating between haploid cells is a prerequisite for infection in *Microbotryum*, and thus each newly diseased plant represents the completion of a sexual reproductive cycle. In heterothallic fungi, mating can occur only between haploid cells carrying different mating-types, which are controlled by alleles at one or two loci [23, 24]. In a few fungi, recombination suppression has evolved around the mating-type locus/loci [25–27], sometimes leading to structurally dimorphic chromosomes similar to the sex chromosomes in plants and animals [28, 29]. The suppression of recombination on mating-type chromosomes is expected to lead to degeneration; this can be manifested as transposable element accumulation [30], codon usage degeneration [31], accumulation of deleterious mutations [32], and eventually to chromosomal dimorphism, and thus the emergence of allosomes [12]. Such degeneration has in fact been observed on the mating-type chromosomes of some fungi, including the ascomycete *Neurospora tetrasperma* [31] and the basidiomycete *Microbotryum lychnidis-dioicae* [12, 30, 33], the most well-studied representative of the anther-smut fungi.

We sequenced the genome of haploid isolate Lamole p1A1 [11], of the a_1_ mating type, to represent the *M. lychnidis-dioicae* species found in association with the perennial, dioecious host, *Silene latifolia.* RNA-Sequencing of distinct life cycle stages was incorporated to validate gene content and measure expression changes during infection. We identified gene family expansions that could play a role in plant infection by comparing *M. lychnidis-dioicae* to other basidiomycetes, taking advantage of increasing genome coverage of the Pucciniomycotina subphylum. The identification of genes that are induced or repressed during infection highlighted carbohydrate active enzymes (CAZymes) that may be involved in host cell degradation or manipulation of host development. Additionally, the *M. lychnidis-dioicae* genome is riddled with a diverse array of transposable elements (TEs), including a higher proportion of Helitron elements than found in the much larger and more highly-repetitive genomes of related rust fungi. Genome regions corresponding to the mating-type chromosomes of *M. lychnidis-dioicae* [11] are enriched for repetitive sequence. Further analysis of the sequence of the entire *a*_*1*_ mating-type chromosome identified more than 300 genes linked with mating-type. Together, these findings provide an in-depth portrait of genetic architecture and adaptation in a specialized fungal plant pathogen.

## Results

### Genome sequence and content

The 25.2 Mb haploid genome of *M. lychnidis-dioicae* was sequenced using 454 technology, generating high coverage of three different-sized libraries (Additional file 1), and assembled using Newbler (Table 1). The assembly was comprised of 1,231 scaffolds where the average base was present in a scaffold of 185 kb and a contig of 50 kb (N50 measure, Table 1). Despite the large number of contigs, the assembly was a nearly complete representation of the sequenced genome, comprising 97 % of sequenced bases. The assembly included five scaffold ends with the typical fungal telomere repeat (TTAGGG), though three of these scaffolds were smaller than 1 kb in size.

**Table 1:** Genome statistics of nuclear genome and mating-type chromosome regions

	Nuclear genome	NRR^a^ regions	PAR^b^ regions
Assembly size (Mb)	26.1	1.86	0.38
Scaffolds (count)	1,231	85	2
Scaffold N50 (kb)	185	48	381
Contigs (count)	2,104	229	16
Contig N50 (kb)	50	13	45
GC content (%)	55.4	54.6	53.9
TE content (%)	14	41	13
Protein coding genes	7,364	350	99
Mean coding length	1,614	1,344	1,408
Median coding length	1,338	954	1,302
Mean exons/gene (count)	5.6	4.6	5.1
Mean intercds length (bp)^c^	1,181	2,600	1,861
tRNAs	134	5	2

^a^Non-recombining regions (NRR). ^b^Pseudo-autosomal regions (PAR). ^c^average length between coding sequence (cds) start and stop

High coverage strand-specific RNA-Seq, generated from three biological conditions (Additional file 2) assisted with the prediction of 7,364 protein coding genes and identified expression changes potentially important for the pathogenic lifecycle. Sampled conditions included two *in vitro* conditions, haploid cells grown on yeast peptone dextrose media (YPD) agar (referred to as rich) or on 2 % water agar (referred to as nutrient limited). These were compared to a sample from infected male plant tissue during the late stages of fungal development, where teliospores form on partially and fully opened smutted flowers [34, 35] (referred to as “MI-late”). Incorporation of RNA-Seq into predicted gene structures (Methods) defined UTRs for the vast majority (more than 6,100) predicted genes; the average length of 5′ and 3′ UTRs was 183 bases and 253 respectively. Coding sequences average 1,614 bases (median of 1,338 bases) in length and contain 5.6 exons; genes are separated by intergenic regions 502 bases in length on average. The *M. lychnidis-dioicae* gene set has high coverage of a core eukaryotic gene set [36], highest in fact than any of the fungal gene sets used in comparative analysis (Additional file 3), suggesting the assembly includes a highly complete gene set.

The mitochondrial genome consisted of a single finished contig of 97 kb and included the canonical set of genes. These were the respiratory-related proteins of the NADH dehydrogenase family (*nad1-6* and *nad4L*), apocytochrome b (*cob*), cytochrome oxidases (*cox1-3*), the proteins related to ATP synthesis (*atp6*, *atp8* and *atp9*); ribosomal RNAs (*rns*, and sequence similar to *rnl*); ribosomal proteins (*rps3*); DNA polymerase (*dpo*) and 25 tRNAs. Homing endonucleases of the LAGLIDADG and GIY-YIG families and a maturase protein were located in mitochondrial intronic regions, including within three introns of *cox1*.

To examine the presence of AT-rich isochores and more generally the genome structure in GC composition in *M. lychnidis-*dioicae, we measured the fluctuation of GC percent along the assembly. Although the genome is not organized in discrete isochores as in some fungal genomes [37], we observe some large-scale variation (>100 kb) in base composition within chromosomes, as well as finer-scale fluctuations (Additional file 4A). The GC content was positively correlated with coding density, with the most significant correlations for 5 and 10 kb windows explaining 16.8 % of the variance (Additional file 4B). This correlation has been explained in other systems by biased codon usage toward GC-rich codons or alternatively biased gene conversion occurring more frequently in coding than in non-coding sequences [38]. In fact, an analysis of the preferred codons (*i.e.,* the most frequently used codons in the predicted genes), showed that 17 out of 18 had a GC base in the third position, which is the most degenerate position and therefore primarily influences the GC composition of genes (Additional file 5).

### Shifts in transposable element type, location, and impact of RIP

The genome of *M. lychnidis-dioicae* contained diverse transposable elements (TEs), represented by 286 consensus elements, covering 14 % of the total assembly. The overall TE content was lower than of other species in Pucciniomycota; in *Puccinia graminis* f. sp. *tritici* or *Melampsora larici-populina*, in which TEs account for nearly 45 % of the assembled genomes, contributing to the expanded genome size of these fungi [39]. Among class I retrotransposon elements (36 % of TE sequences), Long Terminal Repeat (LTR) elements were the most common (28 % of TE sequences), in particular *Copia*-like elements (20 % of TE sequences), in agreement with prior studies of genome sampling and expressed sequence profiles [30, 40]. The remainder of class I elements consisted of Long Interspersed Nuclear Element (LINE) (7 % of TE sequences) and *Dictyostelium* Intermediate Repeat Sequence (DIRS) elements (1 % of TE sequences). Among class II DNA transposons (23 % of TE sequences), Terminal Inverted Repeat (TIR) and *Helitron* elements, which transpose by rolling-circle replication [41] account for 12 % and 10 % of TE sequences, respectively (Additional file 6, Table 2). The *Helitron* proportion was an order of magnitude higher than in the more repetitive genomes of other Pucciniomycota fungi *P. graminis* f. sp. *tritici* and *M. larici-populina*, for which a similar analysis characterized only 1 % of TE elements as *Helitrons* [39].

**Table 2:** Genome coverage of TE families (10,283 copies of 286 REPET consensus sequences)

TE copies	Copy number	Coverage relative to TE space		Coverage relative to assembly size	
LTR	1018	27.70	Class I 35.64	3.89	Class I 5.01
DIRS	57	1.34		0.19	
LINE	392	6.61		0.93	
SINE	0	0		0	
TIR	438	12.18	Class II 22.76	1.71	Class II 3.20
MITE	39	0.39		0.06	
Helitron	373	10.18		1.43	
Unknown	1942	41.60	41.60	5.85	5.85
Total	4259	100	100	14.06	14.06

The TE categories varied significantly in their proximity to genes. A chi-squared test of heterogeneity found a significant difference in the TE content of regions nearby genes, comparing regions less than versus greater than 1 kb upstream and downstream of genes (upstream region p-value < 2.2e-16; downstream region p-value < 2.2e-08, Additional file 7). In particular, class II elements (TIR and *Helitrons*) were closer to genes than class I elements (LTR and LINE), with a greater enrichment upstream of genes compared to downstream (Additional file 7). In addition, there appeared to be an association between TE-rich regions in *M. lychnidis-dioicae* and genes for Small Secreted Proteins (SSPs), which can include effector proteins involved in host-pathogen interactions as suggested in some pathogen genomes [42, 43]. SSPs were indeed located nearer to TEs than the set of all other non-SSP genes (Chi-squared p-value < 5e-4; Additional file 8).

Hypermutation in TEs that resembles the genome defense, Repeat-Induced Point mutation (RIP), has previously been observed in the LTR elements (*copia*-like and *gypsy*-like elements) and *Helitron* transposons of *M. lychnidis-dioicae* [44, 45]. Some genes that appeared similar to those necessary for RIP in *Neurospora crassa* [46, 47] were found in the *M. lychnidis-dioicae* genome. These include a cytosine methyltransferase (MVLG_04160), but establishing the orthology with the RIP-essential *rid* gene from *N. crassa* versus other cytosine methyltransferases (*e.g.* Dim-2) would require further investigation. *M. lychnidis-dioicae* sequences similar to the Dim-5H3 histone methyltransferase that is essential for marking RIP regions in *N. crassa* (MVLG_02125, MVLG_05378) were also found.

Evaluation of dinucleotide signature at transition mutations in 179 TE families (2,298 genome copies, Additional file 9) revealed that 40 % of these TE copies exhibited elevated substitution rates that were particular to which nucleotide was 3′ to the cytosine (Methods, Additional file 10). Eighty per cent of TE copies with high frequencies of cytosine mutation showed a bias toward CpG dinucleotides, consistent with the “CpG effects” [48] of maintenance methylation known in eukaryotes [49–51], including fungi [45, 52] where CpG methylation of TEs have been shown in ascomycete and basidiomycete fungi [53]. Notably, the rate of CpG mutations varied according to TE order and superfamily examined: TE copies exhibiting a pattern of frequent transition at CpG sites appeared lower for class II DNA transposons (*Helitron*-type and TIR elements) than that of class I Retrotransposons (21 % and 58 % respectively) (Additional file 10). In addition, the GC content in TEs (54.4 %) was very close to GC content in genes (56.0 %) and genome (55.4 %).

In addition, the *M. lychnidis-dioicae* genome was found to contain the core RNAi machinery components, that may act to constrain proliferation of transposable elements, contrasting with some other Basidiomycetes that have lost this pathway [54]. The RNAi pathway components were identified based on similarity to known RNAi genes in fungi and validated by examining predicted functional domains (Methods). The genome of *M. lychnidis-dioicae* was also found to contain one copy of a RNA-dependent RNA polymerase (MVLG_02137), two copies of Argonaute (MVLG_06823 and MVLG_06899), and one copy of Dicer (MVLG_01202). All of these components of RNAi machinery were expressed under each of the conditions examined, suggesting the pathway is active across life cycle states, although one copy of Argonaute (MVLG_06823) was significantly more highly expressed (corrected p-value <0.01) during infection compared to nutrient limited and rich agar media.

### Mating-type locus and chromosome

The central proteins involved in mating-type determination in basidiomycetes were found in *M. lychnidis-dioicae*. Orthologs of the two-component homeodomain transcription factor that functions in post-mating compatibility, HD1 (MVLG_07149) and HD2 (MVLG_07150), were assembled in a 14.1 kb region; as previously described HD1 and HD2 are adjacent and divergently transcribed in *M. lychnidis-dioicae* [55], similar to other fungi [56]. Both the mating pheromone receptor [57] and the homeodomain compatibility factor identified above are located at the ends of their respective supercontigs, such that the genomic proximity of these two essential mating-type-determining loci is unclear. The chromosomes bearing these two mating-type loci show suppressed recombination across most of their length, with only two small recombining regions at their ends, *i.e.,* pseudo-autosomal regions (PARs) [11, 12, 33, 55, 58]. The assembly scaffolds corresponding to the non-recombining regions (NRRs) and to the PARs were identified based on alignment to an optical map of the mating-type chromosomes [12, 33] and by performing additional sequencing of gel purified chromosomes (Methods). A total of 449 genes mapped to the a_1_ mating-type chromosome, including 350 genes found on the NRRs and 99 genes found on the PARs (Table 1, Additional file 11). Other than the genes for the pheromone receptor, the homeodomain transcription factors, and the STE20 protein kinase, no other genes on the mating-type chromosome have a predicted function linked to mating in other systems.

**Fig. 2:**
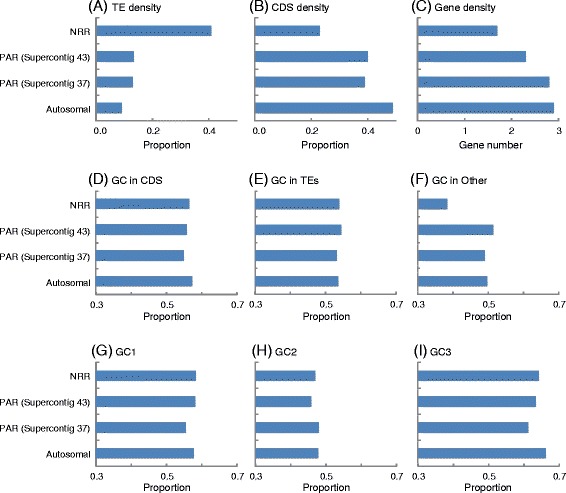
Comparisons of sequence characteristics in non-recombining regions (NRR) of the mating-type chromosome, pseudoautosomal regions (PAR), and autosomes. The genomic regions are shown, with results for the two supercontigs (“37” and “43”) corresponding to the two PARs presented separately. **a** Transposable element (TE) density is shown as the total length of TE sequences over the total length of DNA analyzed. **b, c** Gene density is shown as the proportion of the total length of coding region (CDS) over the total length of DNA analyzed, and number of putative genes identified per 10000 nucleotides, respectively. **d–f** Proportion GC base pair contents are shown for CDS, TEs, and for the remaining, predominantly intergenic regions. **g–i** Proportion GC base pair content for protein-coding genes are shown relative to first-, second-, and third-codon positions (“GC1”, “GC2” and “GC3,” respectively)

Consistent with the expectation in regions of suppressed recombination (*e.g.*, [59]), the TE density (Fig. 2a) was several fold higher in the non-recombining region (NRR) of the *a*_*1*_ mating-type chromosome (41 %) relative to the autosomes (9 %), confirming prior studies of a TE accumulation on the mating-type chromosomes as a whole [11, 12, 33]. The pseudoautosomal regions (PARs, supercontigs 37 and 43) displayed a TE content (~13 %) more similar to the estimate for autosomal regions than to the NRR of the mating-type chromosome. Gene density in the NRR of the mating-type chromosome (23 %), estimated as CDS density, was less than half the gene density of the autosomes (49 %) (Fig. 2b). The number of genes predicted per 10 kb positions also indicated a lower density in the NRR of the mating-type chromosome (1.7 genes) than in the autosomal partition (2.9 genes) (Fig. 2c). As with TE content, the PARs displayed gene density values (~40 % for CDS density and ~2.5 for genes per 10 kb) closer to the autosomal estimates than the NRR of the mating-type chromosome. The NRR of the mating-type chromosomes contained genes of the same distribution, with the exception of 2-fold elevated density of small secreted proteins (SSPs) and, in particular, a 5-fold enrichment of Cys-rich SSPs compared to the autosomes.

With regard to base pair composition, again the NRR exhibited a pattern distinct from the PARs or autosomes in GC content. The GC content in protein coding genes, irrespective of codon position, were similar among the NRR of the mating-type chromosome, PAR, and autosomes (Fig. 2d), as were the contents represented by TEs (Fig. 2e). However, in other sequences, representing inter-genic regions not consisting of TEs, the NRR displayed markedly reduced GC content (Fig. 2f) while PARs and autosomes had similar GC levels. GC content of codon positions within protein-coding genes did not vary among the NRR, PARs, and autosomes (Fig. 2g–i), notably in that the third-codon position patterns was not reflective of intergenic GC composition variation across regions. This pattern as well as the lower GC content observed for the second-codon position compared to first or third positions is consistent with some prior research [60]. The correspondence analysis of codon usage (Additional file 12) among the non-recombining region of the mating-type chromosome, PAR and autosomal did not indicate obvious differences in codon usage.

### Gene conservation and lineage-specific changes

**Fig. 3:**
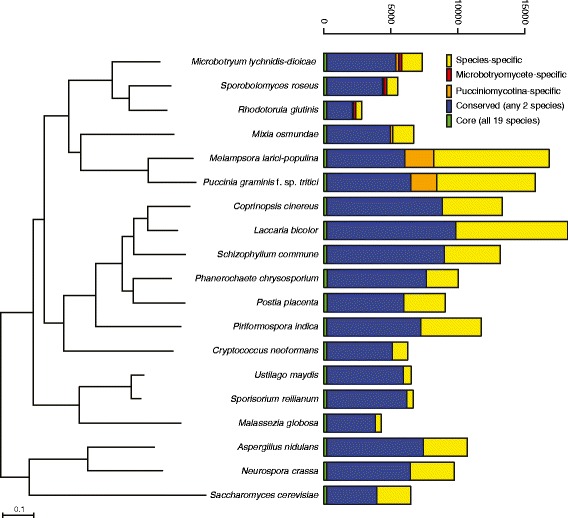
Phylogenetic relationship and gene conservation of *Microbotryum lychnidis-dioicae* and 18 compared fungi. Phylogeny (left panel) is based on concatenated MUSCLE alignments of 80 single copy genes. Species phylogeny was inferred using RAxML (PROTCATWAG model) with 1,000 bootstrap replicates; all nodes were supported by at least 99 % of replicates. Ortholog conservation (right panel) highlights genes conserved in all species (core, green), genes conserved in at least two species (conserved, blue), genes unique to the Pucciniomycotina or Microbotryomycetes (orange and red, respectively) and genes unique to a given species (species-specific, yellow)

The comparison of 7,364 predicted proteins of *M. lychnidis-dioicae* to those of diverse basidiomycetes revealed gene loss and gain patterns relevant in terms of the growth and pathogenesis of this organism. We included representatives of the three subphyla of basidiomycetes (Pucciniomycotina, Agaricomycotina, and Ustilaginomycotina), as well as three Ascomycota species as outgroups (Fig. 3, Additional file 13). Within the Pucciniomycotina, species compared included *M. lychnidis-dioicae* and two closely related Microbotryomycetes (*Sporobolomyces roseus* and *Rhodotorula glutinis*) and three other more distantly related species; within this group the two other plant pathogens (*P. graminis* f. sp. *tritici* and *M. larici-populina*) are biotrophic, like *M. lychnidis-dioicae*. These comparisons revealed 2,451 *Microbotryum* gene clusters representing 2,613 genes that were broadly conserved in the Basidiomycota (present in at least 13 of the examined 15 other basidiomycete genomes). The gene families specific to Pucciniomycotina, with orthologs present only in *M. lychnidis-dioicae* and/or the other Pucciniomycotina species, (Fig. 3, orange boxes) was composed of a small set of 224 predicted proteins from *M. lychnidis-dioicae*; the two rusts (*P. graminis* and *M. larici-populina*) share a larger number of Pucciniomycotina-specific proteins in part due to their expanded genome size. Examining proteins conserved across the Microbotryomycetes, orthologs of 4,844 *M. lychnidis-dioicae* proteins were present in at least one other species, and of these 233 were specific to the Microbotryomycetes. A set of 190 gene duplications occurring specifically along the *M. lychnidis-dioicae* lineage were identified using phylogenetic analysis (Additional file 14, Additional file 15, phylomedb.org); these did not display functional enrichment for gene ontology (GO) term assignments, as GO terms were only assigned for 19 of the 190 genes. While most genes (70 %) were shared with occurrence in at least one other species, the remaining set of 1,534 genes appear specific to *M. lychnidis-dioicae*.

The identification of enriched or depleted PFAM domains for *M. lychnidis-dioicae* compared to other fungi revealed significant differences in functional categories between these genomes. A total of six protein domains were significantly enriched or depleted (*q*-value < 0.05) in *M. lychnidis-dioicae* compared to the other basidiomycetes examined (Table 3, Additional file 16). Enriched domains include secretory lipases, and two domains of unknown function, DUF23 (glycosyl transferase 92) and DUF1034 (Fn3-like). DUF23 (PF01697) was present in five copies in *M. lychnidis-dioicae* and was only present otherwise in *Mixia osmundae* and *S. roseus* in our comparison; three of these five genes were mapped to the GT2 CAZY family expanded in *M. lychnidis-dioicae* (see below). Both *M. lychnidis-dioicae* and the rusts contain a large number of proteins with the DUF1034 domain; 8 of the 10 *M. lychnidis-dioicae* proteins with a DUF1034 domain also contained a subtilase family protease domain. Phylogenetic analysis of proteins with the DUF1034 domain suggested that independent gene family expansions occurred in different species; the rusts formed a separate clade from *Microbotryum* that is further subdivided, mostly along species lines (Additional file 17). Domains depleted in *M. lychnidis-dioicae* relative to other basidiomycetes were also identified, including Cytochrome p450, NACHT, and F-box domains (Table 3, Additional file 16). A more narrow comparison of the three Microbotryomycetes to the other species within the Pucciniomycotina identified additional expansions and depletions common to the species in this lineage. A fungal trichothecene efflux pump (PF06609) gene family was enriched in *M. lychnidis-dioicae* and *S. roseus*, with 16 copies in each genome (Table 3). By contrast, the alpha-kinase family that is highly expanded in the rusts [39] was absent in the Microbotryomycetes. The cutinase domain shows a similar conservation pattern; while multiple cutinase genes are found in other biotrophic pathogens, no copies were detected in *M. lychnidis-dioicae*. At lower levels of significance, the CBM1 cellulose binding domain was detected as absent from all species in the Pucciniomycotina with the exception of *R. glutinis* (Table 3, Additional file 16). More specific analysis of these enriched and depleted domains is presented below.

**Table 3:** Expanded or depleted PFAM domains in *Microbotryum lychnidis-dioicae*

PFAM domain	*M. lychnis-dioicae*	*S. roseus*	*R. glutinis*	*M. osmundae*	*M. larici-populina*	*P. graminis-tritici*	*S. reilianum*	*U. maydis*	*M. globosa*	Agaricomycetes^c^ (7)	Basidiomycete comparison^a^		Pucciniales comparison^b^	
											*p*-value	*q*-value	*p*-value	*q*-value
PF00067.15 Cytochrome P450	10	7	5	14	29	17	15	20	6	95	1.43E-11	6.49E-08	1.28E-02	1
PF05729.5 NACHT	1	2	1	1	1	1	1	1	0	44	6.20E-08	1.41E-04	4.51E-01	1
PF01697.20 Glycosyltransferase family 92	5	1	0	1	0	0	0	0	0	0	1.65E-05	2.50E-02	1.99E-02	1
PF06280.5 Fn3-like (DUF1034)	10	0	0	0	5	9	1	1	0	1	2.54E-05	2.88E-02	1.00E + 00	1
PF00646.26 F-box	7	18	12	12	9	11	11	10	3	43	5.20E-05	4.33E-02	2.72E-02	1
PF03583.7 Secretory lipase	7	0	0	0	0	0	3	2	6	0	5.72E-05	4.33E-02	1.76E-03	3.07E-01
PF00734.11 CBM1 Fungal cellulose binding	0	0	3	0	0	0	0	0	0	21	1.21E-04	7.10E-02	6.60E-02	1
PF02816.11 Alpha kinase	0	0	0	0	79	39	0	0	0	5	1.25E-04	7.10E-02	2.80E-27	1.27E-23
PF07690.9 MFS1 Major Facilitator Superfamily	119	110	26	64	90	72	104	98	30	129	4.11E-02	1	2.06E-08	4.67E-05
PF01753.11 zf-MYND finger	7	11	26	3	7	4	2	2	1	41	4.61E-04	2.33E-01	5.16E-08	7.82E-05
PF01083.15 Cutinase	0	0	1	4	21	9	3	4	0	2	7.90E-02	1	6.49E-07	5.89E-04
PF01670.9 Glycosyl hydrolase family 12	0	0	0	10	14	3	0	0	0	1	1.70E-01	1	1.08E-06	6.56E-04
PF11327.1 DUF3129	0	0	0	4	13	10	0	0	0	1	2.62E-01	1	1.08E-06	6.56E-04
PF00097.18 Zinc finger, C3HC4 type	23	11	9	26	22	93	28	24	12	24	6.82E-01	1	1.16E-06	6.56E-04
PF00080.13 Copper/zinc superoxide dismutase	0	0	0	2	6	18	0	0	0	1	2.62E-01	1	1.94E-06	9.79E-04
PF00098.16 Zinc knuckle	10	6	1	6	11	59	8	7	5	11	7.63E-01	1	6.31E-06	2.87E-03
PF06609.6 Fungal trichothecene efflux pump	16	16	5	6	6	3	8	9	2	12	4.10E-02	1	1.18E-05	4.86E-03
PF12013.1 DUF3505	0	0	0	1	4	16	0	10	0	0	2.65E-01	1	2.15E-05	8.14E-03
PF03101.8 FAR1 DNA-binding domain	0	0	0	1	14	4	1	1	0	1	4.09E-01	1	6.65E-05	2.32E-02
PF00083.17 Sugar (and other) transporter	54	56	13	31	46	34	56	46	14	61	3.28E-01	1	1.71E-04	5.54E-02
PF07738.6 Sad1/UNC-like	2	1	2	3	24	7	2	1	2	2	5.87E-01	1	2.45E-04	7.42E-02

^a^M. lychnis-dioicae compared to all other Basidomycetes; ^b^ Microbotryales (M. lychnis-dioicae, S. roseus, R. glutinis) compared to other Pucciniales (M. larici-populina, P. graminis tritici, M. osmundae); ^c^Agaricomycetes represent average of the 7 species in this group; see Additional file 11 for counts per species

The expansion of the secretory lipases appeared specific to *M. lychnidis-dioicae* within the Pucciniomycotina (Table 3). Among all other basidiomycete genomes compared, secretory lipases are also highly represented in *Malassezia globosa* (Ustilaginomycotina)*,* a skin fungus associated with human dandruff and dependent on its host for lipids. *Malassezia globosa* has an additional gene family expansion associated with lipid acquisition; this species has 6 copies of phospholipase C, whereas *M. lychnidis-dioicae* contains only a single phospholipase C protein. Unlike *M. globosa*, *M. lychnidis-dioicae* does not depend on lipids for growth, and contains a predicted fatty acid synthase (MVLG_04698). The secretory lipase family is also present at lower copy number in the two Ustilaginomycotina corn smuts, *Sporisorium reilianum* and *Ustilago maydis*, of which the latter responds to lipids, including corn oils, as part of a developmental switch [61]. A phylogenetic analysis of the secretory lipases in this comparison revealed that the *M. lychnidis-dioicae* lipases have undergone a lineage specific expansion, as in *M. globosa* (Fig. 3a). Most lipases were predicted to be secreted including three of the seven *M. lychnidis-dioicae* lipases. However four of the seven genes appeared partial based on alignment of the protein sequences; the missing 5′end from two genes deleted the region containing a secretion signal in paralogous copies. Further refinement of the assembly or transcripts is needed to identify the full length version of these genes or confirm if they are perhaps pseudogenes (see below), and establish their relative location in the genome.

**Fig. 4:**
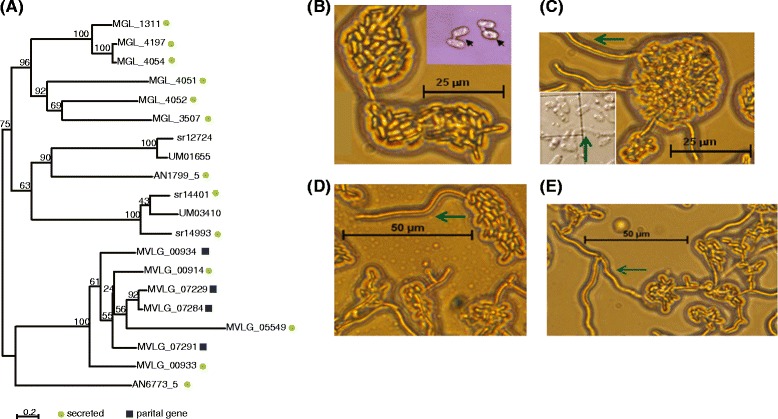
Expansion of lipase gene family in *Microbotryum lychnidis-dioicae* and *Malassezia globosa*. Both *M. lychnidis-dioicae* and *M. globosa* contain a higher number of proteins with a lipase domain (PF03583.7) relative to the other fungi examined. **a** Phylogenetic tree of proteins with this domain. Protein sequences were aligned with MUSCLE, and a phylogeny was inferred from this alignment using RAxML (PROTCAT model, DAYHOFF matrix). A total of 1000 bootstrap replicates were performed, and the percent of replicates shown is on the tree nodes. All *M. lychnidis-dioicae* proteins are indicated by their respective MVLG designation. Similar lipase genes from other species are included, with the gene prefix denoting the species as follows: AN, *Aspergillus nidulans*; UM, *Ustilago maydis*; sr, *Sporisorium relianum*; MGL, *Malasezzia globosa*. Scale corresponds to substitutions per site. **b–e** Treatment of mated *M. lychnidis-dioicae* cells with various lipids. **b** water-treated mated cell control (Inset: higher magnification showing cells with conjugation bridge, black arrows); (**c**) mated cells treated with commercially-available corn oil (Inset: higher magnification of filamentation); D, mated cells treated with α-tocopherol; (**e**), mated cells treated with phytol. The green arrows show areas of filamentation emanating from mated cells after treatment with each specific type of lipid. **b** and **c** size bars, 25 μm; (**d**) and (**e**), size bars, 50 μm

Previous work in *M. lychnidis-dioicae* has shown that mating mixtures of haploid cells produce hyphae in response to phytols and to tocopherols [62]. We therefore exposed haploid sporidial cells (p1A1 or p1A2 strains) or mated cells (p1A1 and p1A2 mixed together) to various lipids or oils, including commercially available corn oil, (+/−)-α-tocopherol, and phytol. Each stimulated filamentous growth of mated mixtures (Fig. 4b–e), but had no observable effect on haploid cells (unmated). The observation that mated cells also respond to corn oil supports the hypothesis that lipid response may be important for the development of this species. This is possible, as lipids are likely present on the host meristem [63]. Moreover, we find that at least three lipase genes are differentially expressed when exposed to phytols (a constituent of chlorphyll), compared to similarly treated cells in the absence of phytols (see below; Additional file 18).

An important group of proteins identified as being expanded or depleted in *M. lychnidis-dioicae* are those predicted to be involved in cell wall modifications, both in terms of fungal cell walls, but also as might affect host plants. Families of structurally-related carbohydrate catalytic and carbohydrate-binding modules (or functional domains) are described in the CAZy database (www.cazy.org) [64]. Such enzymes break down, modify, or build glycosidic bonds. The assignment of the predicted proteins derived from a genome to CAZy families helps to shed light on the particular glycobiological features of an organism [65]. A total of 236 *M. lychnidis-dioicae* protein models were mapped to protein families in the CAZy database based on sequence conservation (percentage identity over CAZy domain length) (Additional file 19). The CAZy family profile of *M. lychnidis-dioicae* was then compared to that recently published for 33 basidiomycetes [66], in order to identify expanded and reduced families (Table 4, Additional file 20).

**Table 4:** Selected CAZY expansions and depletions

	GT total	GH total	Beta glucan modification^a^	Cellulose related^b^	Xylan related^c^	GH26 beta-mannanases	Pectin/pectate lyases
*Microbotryum lychnidis-dioicae*	98	82	4	0	0	4	0
Average basidiomycete^d^	69.5	179.7	28.9	37.3	14.0	0.5	2.9
*Piriformospora indica*	65	194	31	98	41	1	12
*Ustilago maydis*	58	100	26	4	8	1	1
*Puccinia graminis f. tritici*	81	154	11	15	5	4	4
*Melampsora laricis-populina*	84	169	14	23	9	3	4

^a^GH16,GH72,GH81,GH128. ^b^GH6, GH7, GH8, GH9, GH12, GH44, GH45, GH131, AA9, and CBM1. ^c^GH10, GH11, GH30, GH29, GH95, GH51, GH115, GH74. ^d^Average count for 33 basidiomycete genomes; see Additional file 20

With 98 candidate glycosyltransferases (GTs), *M. lychnidis-dioicae* has more than any of the 33 basidiomycetes used in the comparison (average = 69.5; min = 41; max = 95). Both *M. lychnidis-dioicae* and *Puccinia graminis* contain a candidate fucosyltransferase, which is not present in the other basidiomycetes surveyed. This is compatible with the known presence of fucose in the cell wall of *Microbotryum* [67, 68]. Two other families expanded in *M. lychnidis-dioicae* include alpha-mannosyltransferases (GT32 and GT62), suggesting that the cell wall includes a larger fraction of alpha-mannan than in other species. In fungi, the synthesized cell wall carbohydrates are frequently remodeled by the action of dedicated glycoside hydrolases and transglycosidases that are found in distinct CAZy glycosyl hydrolase (GH) families. A notable feature of *M. lychnidis-dioicae* is that it has a reduced number β-1,3-glucan cleaving or modifying enzymes of families (GH16, GH72, GH81, and GH128).

Examination of the other GH families and of the other categories involved in carbohydrate breakdown (PL, CE, AA and ancillary CBM) revealed that *M. lychnidis-dioicae* completely lacked cellulases (no GH6, GH7, GH8, GH9, GH12, GH44, GH45, nor any GH5 with highest sequence similarity to characterized cellulases) (Table 4, Additional file 20). In addition no cellulose-targeting lytic polysaccharide monooxygenase of family AA9, nor any broad specificity β-glucanase of family GH131, nor any cellulose-binding module of family CBM1 could be found. This clearly shows that *M. lychnidis-dioicae* does not interact with nor digests cellulose during its interaction with plants, a finding confirmed by the failure of *M. lychnidis-dioicae* to grow on cellulose as a sole carbon source (Additional file 21). *Microbotryum lychnidis-dioicae* also completely lacks xylanase (no GH10, GH11, GH30), xyloglucanase (no GH74), and the enzymes for the cleavage of side-chains of xylan, xyloglucan and rhamnogalacturonan (no GH29, GH95, GH51, GH115), indicating that these cell wall polymers are not a carbon source for the fungus (Table 4, Additional file 20). Consistent with this prediction, *M. lychnidis-dioicae* failed to grow on xylan as a sole carbon source (Additional file 21).

*Microbotryum lychnidis-dioicae* was able to grow on pectin as a sole carbon source (Additional file 21), although it does not break down pectin by the action of pectin/pectate lyases, as these enzymes are also absent from the genome (Table 4, Additional file 20). Instead, the genome harbors a suite of six family GH28 enzymes, which cleave polygalacturonic acid after its methylester groups have been removed by the action of six family CE8 pectin methylesterases. This CE8 family is present at high numbers in the two rust fungi and *M. lychnidis-dioicae*; the copy number amplification in *M. lychnidis-dioicae* appears to be due to tandem duplication, with one array of two genes and a second array of four genes. Four of the six CE8 copies have a predicted secretion signal, supporting a potential role in interacting with the host plant. Compared to 33 other basidiomycetes, *M. lychnidis-dioicae* stands out in having a significant expansion of its enzymatic arsenal for the breakdown of β-mannan, a polysaccharide present throughout plants but more abundant in flowers, siliques and stems [69]. *Microbotryum lychnidis-dioicae* encodes four candidate β-mannanases of family GH26 and a comparison with biochemically characterized enzymes shows that 10 out of its 19 GH5 enzymes also target β-mannan (the other *M. lychnidis-dioicae* GH5 enzymes target β-1,3-glucans (5 proteins), glucocerebrosides (3 proteins) and β-1,6-glucan (1 protein)). This β-mannan digestion arsenal is augmented by the presence of a GH2 enzyme, which shows a strong relatedness to characterized β-mannosidases.

Homogalacturonan is a major component (60 %) of plant pectin and the degradation pathway is required in several stages of plant development. One of these stages is anther dehiscence when pollen grains are released; this process requires pectinesterases and polygalacturonases. As indicated above, a total of six CE8 family pectin methylesterases are found in *M. lychnidis-dioicae*, of which four are predicted secreted proteins (MVLG_02682, 04072, 04073, 4074). Part two of the pathway requires polygalacturonase; a total of six *M. lychnidis-dioicae* proteins contain the polygalacturonase GH28 (PF00295) domain, of which MVLG_02498 is highly induced (over 1,000 fold) in MI-late. The homogalactorunan degradation pathway of *M. lychnidis-dioicae* may thus perform a similar role as pollen in anther dehiscence when the flowers bloom, since during teliospore formation of *M. lychnidis-dioicae*, host pollen is no longer available to perform that function.

Multiple classes of transporters are expanded in *M. lychnidis-dioicae* (Table 3), enabling uptake of diverse substrates. Major facilitator transporters, sugar transporters, and the fungal trichothecene efflux pump are present at high copy number relative to other Pucciniomycotina. A fungal trichothecene efflux pump, *TRI12*, was first described in *Fusarium sporotrichioides* as part of the gene cluster involved in trichothecene biosynthesis [70]; trichothecenes are a group of mycotoxins produced by various species of fungi. As the TRI12 domain is present at high copy number in *M. lychnidis-dioicae* and other Basidiomycetes are not known to produce trichothecenes, this suggests that this domain may have a role in transporting other small molecules.

Sugar transporters also play an important role in virulence of biotrophic plant pathogens, such as *Ustilago maydis* and several species of rust fungi. Specifically, a plasma membrane-localized sucrose transporter (Srt1) in *U. maydis* facilitated direct utilization of sucrose, thus eluding the plant defense mechanism [71]. The HeXose Transporter 1 (Hxt1) gene in the rust fungus *Uromyces fabae* is localized to haustoria to take advantage of that structure for sugar uptake [72]. The *M. lychnidis-dioicae* genome contains a total of 26 potential sugar transporters, with multiple high identity matches to Srt1 and Hxt1, which may fulfill similar roles.

One additional contrast between *M. lychnidis-dioicae* and the two plant pathogenic rust fungi examined suggests a difference in the relative importance of response to superoxides. Reactive oxygen species (ROS), including superoxides or H_2_O_2_ produced by the host plant, are a canonical part of the defense response to pathogens. *Microbotryum lychnidis-dioicae* is depleted in domains for Peroxidase (PF01328) and copper/zinc superoxide dismutase (PF00080); the two other Microbotryomycetes also lack proteins with these domains. Despite the reduced repertoire of such predicted proteins in *M. lychnidis-dioicae* relative to the rust fungi, six proteins (MVLG_00980, MVLG_03089, MVLG_03931, MVLG_02439, MVLG_03568, and MVLG_04684) were identified as containing peroxidase 2 (PF01328), peroxidase (PF00141), redoxin (PF08534), or Glutathione peroxidase (PF00255) domains. Of these predicted proteins, only MVLG_03089 was differentially expressed under the conditions examined and was up-regulated in MI-late relative to growth *in vitro* in rich medium. In addition, four predicted proteins with iron/manganese superoxide dismutase domains (PF02777 and PF00081), glutaredoxin (PF00462) or catalase (PF00199) domains were found (MVLG_00659, MVLG_06630, MVLG_06939, MVLG_04131). Finally, pathway analysis via MetaCyc predictions (http://fungicyc.broadinstitute.org/) suggests that *M. lychnidis-dioicae* contains components of the glutathione-mediated detoxification pathway: Glutathione transferase (EC 2.5.1.18: MVLG_05985, MVLG_04790) and membrane alanyl aminopeptidase (EC 3.4.11.2: MVLG_03673). However, there appears to be a missing component (3.4.19.9) in this pathway to facilitate formation of an intermediate of a glutathione-toxin conjugate. Biochemical and functional analyses will be required to determine the importance of these predicted enzymes in the ability of the pathogen to survive and flourish in its host.

### Secreted proteins (SP) and candidate effectors

A total of 279 secreted proteins (SPs) were predicted in *M. lychnidis-dioicae* and their expression and conservation examined to identify candidates for interacting with the host (Table 5, Additional file 22). Among the 71 SPs that were smaller than 250 amino acids (small secreted proteins, SSPs), 46 were species specific in our comparative set and further do not share sequence similarity (e-value <1e-3) with any protein in the NCBI protein database. SSPs indeed often appear species-specific, likely because they co-evolve rapidly in an arms race with their hosts [43]. Notably, 48 of the SSPs were significantly up-regulated during plant infection (MI-late compared to rich media), but were not differentially expressed when comparing expression on rich and nutrient limited agar (Table 5), suggesting that these SSPs may play a specific role during plant infection.

**Table 5:** Properties of predicted secreted proteins

Protein length	SP count	Induced in MI late	Repressed in MI late	Induced in water	Repressed in water	FPKM > 1	Highly expressed only in MI late
100	15	4	0	0	0	11	3
150	23	5	0	1	0	20	4
200	15	5	0	2	1	14	
250	18	5	1	1	0	14	3
400	62	8	10	8	3	59	2
500	56	8	7	5	2	55	
600	33	4	2	7	0	33	
700	23	7	1	0	0	23	
800	7	0	2	1	0	7	
900	6	1	0	0	0	6	1
1000	13	2	2	0	1	13	
2000	6	0	1	1	0	6	
3000	2	0	0	0	0	2	
Total	279	49	26	26	7	263	

Several cysteine-rich multigene families were identified among predicted secreted proteins. In some cases these families include tandemly duplicated genes; the MVLG_04105 family contains 4 members predicted to be SSPs (MVLG_04105, 04106, 04107, and 04096), three of which are adjacent in the genome on the mating-type chromosome (see below). Although these proteins lack PFAM domains, two of these are induced during infection. An additional family of Cys-rich proteins with nine members has a subset of six clustered in the genome (MVLG_05513, MVLG_05514, MVLG_05515, MVLG_05533, MVLG_05534, MVLG_05538). Seven of the nine proteins in this family were predicted to be secreted, yet their expression was highly variable, with two up-regulated in nutrient limited conditions and two down-regulated during infection. A small subset of Cysteine-rich proteins contains known protein domains. Two proteins (MVLG_02283 and MVLG_02288) contain the Cysteine-rich secretory protein family domain (PF00188). In addition, a total of 9 proteins contain the fungal-specific Cysteine rich CFEM domain (PF05730). All of these CFEM proteins were predicted to be secreted; four of these were significantly induced and three were repressed in MI-late relative to rich and nutrient limited agar.

To identify genes that could provide a mechanism for linking flower development to fungal development, we compared expression of the predicted secreted proteins with the *S. latifolia* EST library produced from flowers [73]. A total of 37 genes share sequence similarity with *S. latifolia* ESTs; ten secreted proteins matched plant ESTs with at least an e-value of e-19. One *S. latifolia* EST (09F02) showed similarity (BlastX, e-value < 7e-24) with two *M. lychnidis-dioicae* proteins (MVLG_02043 and MVLG_02936); the best match, MVLG_02043, encodes a predicted secreted gamma-glutamyltranspeptidase (GGT). Another EST (33C05) shared sequence similarity with two *M. lychnidis-dioicae* proteins (MVLG_00083, MVLG_02276); these in turn share similarity with the expansin family of “ripening related” proteins and were down-regulated during either growth on nutrient limited agar or late in infection (MI-late). The precise function of plant expansins is poorly defined at the molecular level, and the predicted function of the similar fungal proteins is even less well established. However, one such expansin-related protein in *Laccaria bicolor* was recently found to be expressed specifically in the extracellular matrix (ECM) of symbiotic tissues and localized within the fungal cell wall [74].

Two cysteine-rich secreted proteins (MVLG_02288 and MVLG_02283) matched a *S. latifolia* flower EST annotated as having similarity with the plant PR-1 class of pathogenesis related proteins (PRs); these are proteins defined as encoded by the host plant but induced only in pathological or related situations (possibly of non-pathogenic origin). To be included among the PRs, a protein must be induced upon infection but not necessarily in all pathological conditions [75]. Another *S. latifolia* gene of interest, *SLM2,* is expressed in the stamens of smut infected flowers but not uninfected flowers [22]; four *M. lychnidis-dioicae* proteins showed blast similarity with e-value less than e-6. All of the hits were hypothetical proteins that contained the SRF-type transcription factor domain (PF00319), and three of the four were predicted to be targeted to the nucleus (MVLG_04297, MVLG_06278, and MVLG_07052).

### Response to oxidative environments

Laccase-like multi-copper oxidase proteins are capable of degrading phenolic compounds like polymeric lignin and humic substances [76] and may be involved in the interacton of fungal pathogens with their host plants. Four proteins in *M. lychnidis-dioicae* contain the three multi-copper oxidase (MCO) domains (PF07731, PF07732, PF00394). Two MCO proteins were predicted to be secreted, and another MCO was predicted to have a GPI-anchor to the membrane. The fourth MCO was a membrane-anchored protein (MVLG_03092), with the N-terminus of the polypeptide outside the cell.

The glyoxal oxidase catalyses the oxidation of aldehydes to carboxylic acid and is an essential component of the extracellular lignin degradation pathway of the root rot fungus, *Phanerochaete chrysosporium*. Of a total of seven *M. lychnidis-dioicae* proteins that contain the glyoxal oxidase N-terminal domain (PF07250), two are predicted to be secreted and four have a predicted GPI-anchor. While two of these glyoxal oxidase genes are adjacent in the genome, they do not form a gene cluster with any MCO as observed at the lignin peroxidase gene cluster in *P. chrysosporium*.

### Genes similar to plant hormone synthesis genes

**Fig. 5:**
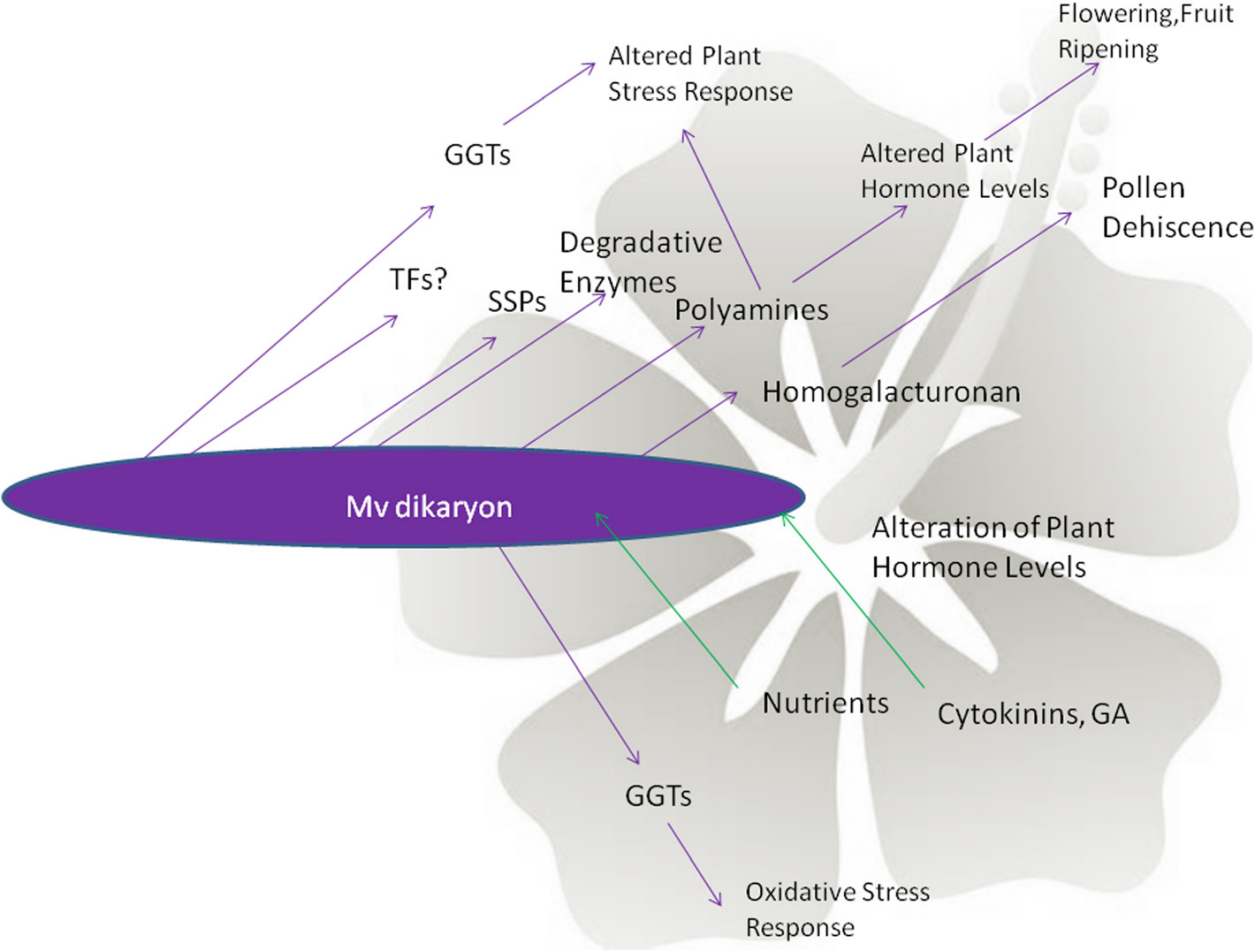
Model of *Microbotryum lychnidis-dioicae* interactions with its host. The potential pathways identified in *M. lychnidis-dioicae* based on inventory of the genome were used to predict products potentially secreted or taken up by the fungus that could affect host development (see text for more detailed description). GA, gibberellic acid

Since *M. lychnidis-dioicae* infection of female *S. latifolia* hosts can alter normal flower development so as to produce pseudomale flowers, we investigated whether the genome might contain genes for pathways that could be associated with such changes. The predicted *M. lychnidis-dioicae* protein database was examined for components of biosynthesis pathways of eight plant hormones (Additional file 23), as well as for other signaling pathways that could have an impact on host gene expression or development. Based on sequence similarity to components for these pathways from plants and microbes, as well as additional confirmation of some complete pathways using MetaCyc predictions (http://fungicyc.broadinstitute.org/), we found evidence that *M. lychnidis-dioicae* encodes enzymes that could participate in hormone biosynthetic pathways, such as polyamine biosynthesis pathways, which produces compounds known to play developmental and stress-response roles in plant physiology [77] (see proposed model in Fig. 5). If these enzymes are, in fact, promoting hormone biosynthesis pathways, one possible explanation is that precursors for these pathways are provided by the plant. Alternatively, the potential components of these pathways we have identified are used by the fungus for functions other than manipulating host development. For example, cytokinin degradation could be mediated by a predicted FAD-oxidase (MVLG_04134), if this enzyme can function as a cytokinin dehydrogenase (EC 1.5.99.12); however, this predicted protein most closely resembles other fungal D-lactate dehydrogenases based on sequence similarity. Similarly, a predicted 2β-dioxygenase (EC1.14.11.13) (MVLG_00840) could be involved in gibberellin inactivation via hydroxylation, although this protein falls more generally into the 2OG-Fe(II) oxygenase superfamily.

Another potential set of pathways for plant signaling involves the production of glycerol lipids, such as diacylglycerol (DAG) or triacylgycerol (TAG). Many studies have demonstrated the importance of compounds like DAG in mammalian signaling [78], and DAG is important in pollen tube elongation in some plant species [79]. Such pathways often involve the action of phospholipase C; a predicted phospholipase C gene (MLVG_07108) and potential phospholipase A (MVLG_03207, MVLG_03384, MVLG_4789) and D (MLVG_01917, MVLG_03610) homologues are found in the genome. Therefore, the organism contains the proteins necessary for synthesizing 1,2-diacylglycerol, 1,2-diacyl-*sn*-glycerol-3-phosphate, 2-lysophosphatidylcholine, and 1-lysophosphatidylcholine. Based on MetaCyc prediction, *M. lychnidis-dioicae* possesses the requisite components of the CDP-diacylglycerol biosynthesis I pathway. In addition, a number of the secretory lipases whose PFAM domain is enriched in *M. lychnidis-dioicae* (PF03583, see above) are implicated in these phospholipase pathways.

### Gene expression changes during infection and in response to lipids

To focus on genes potentially important for infection and interaction with the host, we identified genes whose expression was altered in MI-late. A total of 1,254 genes were differentially expressed in MI-late compared to either rich or nutrient limited agar; of these, a common set of 307 genes were induced in MI-late and 126 were repressed in MI-late compared to both other conditions (corrected p-value <0.001, Additional file 24, Methods). Of the 138 genes in both comparisons with a predicted PFAM domain, transporter domains were most frequently observed; a total of 20 of the 138 functionally assigned proteins corresponded to transporters, with MFS, sugar, OPT, and amino transporters each represented by two or more genes (Additional file 25). Carbohydrate active enzymes, kinases and transcription factors were also highly represented in these MI-late induced genes.

Several different classes of transporters were transcriptionally induced during infection, potentially promoting the uptake of small molecules and nutrients from the host plant. Significant enrichments include domains found in MFS transporters (Fisher’s exact test, corrected *p*-value < 0.001) and sugar transporters (*p* < 0.005), the largest classes of transporters in *M. lychnidis-dioicae* and many fungi. The oligopeptide transporter protein was also enriched at a lower level of significance (*p* < 0.1) and four predicted proteins with this domain (MVLG_03106, MVLG_07217, MVLG_03161, and MVLG_00149) were up-regulated in MI-late. An MFS domain-containing protein of note up-regulated in MI-late was a nitrite transporter (TIGR00886.2; MVLG_00642); this gene is linked to two other genes associated with nitrate assimilation, a nitrite reductase (MVLG_00638) and a nitrate reductase (MVLG_00637). All three genes involved in nitrate assimilation were significantly up-regulated during infection.

In evaluating the expression of the 279 proteins predicted to be secreted, 48 were induced *in planta*. Several cysteine-rich secreted proteins were induced during infection. A pair of linked cysteine rich small secreted proteins (MVLG_04106 and MVLG_04107) was induced during infection. Four other proteins (MVLG_00115, MVLG_00802, MVLG_00815, and MVLG_00859) with the Cys-rich CFEM domain-containing family (PF05730) were significantly induced in infection. These proteins are good candidates for being potential effectors, based on proteins with similar properties that have been shown to be effectors in other systems [80].

Cell wall degrading enzymes may play a role in the infection, particularly during the stage where the fungus causes necrosis of host plant tissue. A total of nine glycoside hydrolases were up-regulated in MI-late; among GH families, GH28 polygalacturonase proteins are mostly highly enriched during infection (*p* < 0.08). Polygalacturonase is required for the second part of the homogalacturonan pathway implicated in pollen dehiscence. MVLG proteins that contain a glyoxal oxidase domain (PF07250) were also significantly enriched (*p* < 0.009) in genes induced during MI-late.

Mated cells of *M. lychnidis-dioicae* respond to corn oil, in addition to the other lipids previously reported [45]. This supports the hypothesis that lipid response may be important for the development of this species. Although we observed no alteration of phenotype for un-mated haploid cells treated with lipids, three predicted cytoplasmic proteins with a secretory lipase domain (MVLG_07229, 07284, and 07291) were highly induced in haploids grown on nutrient limited agar compared to rich media or MI-late infections. To validate the expression levels from RNA-Seq data, relative levels for lipase genes were measured for mated and unmated cells, grown in nutrient limited, rich media, or treated with phytol by qRT-PCR (Additional file 18). Notably, when mated haploid cells were treated with phytol, after 12 h treatment, two genes (MVLG_00914, MVLG_05549) displayed substantial up-regulation, while the remaining three either increased slightly or decreased (Additional file 18). This could reflect priming of cells that are ready to mate for plant cues that would ultimately lead to stable dikaryon formation after successful mating.

## Discussion

This analysis highlights genomic features of *M. lychnidis-dioicae* that reflect its particular lifecycle. *Microbotryum lychnidis-dioicae* grows as a biotroph, similar to the rust fungi, for most of the plant infection cycle. Notably in its capacity as a castrating pathogen, *M. lychnidis-dioicae* also causes necrosis that appears to be limited to developing flowers. Our analysis revealed that gene content contains a different profile than purely biotrophic or necrotrophic plant pathogens. This included a global number of CAZymes that is larger than other biotrophic fungi (Additional file 20); however, at the same time it shows complete loss of many CAZyme families that target the plant cell wall. This is consistent with a primarily intercellular colonization pattern of host apical meristems [81], though it raises questions about how appressorium penetration is accomplished.

Necrotic growth stages may be enabled by the subtilases, laccases, and copper radical oxidases, which are ligninolytic enzymes [82–84]; subtilases, in particular may play a key role in regulating the activities of laccase [76]. By contrast, certain CAZymes present in the biotrophic rust fungi are absent in *M. lychnidis-dioicae*; in particular the absence of cutinases suggests that penetration of the plant surface by *M. lychnidis-dioicae* does not require cutin degradation. This may reflect the fact that the normal portal of entry for infection is via the flower, a tissue that poses less of a barrier to the fungus. Of the plant polysaccharides that constitute a carbon source for many fungi, *M. lychnidis-dioicae* has lost the ability to digest cellulose, xylan, xyloglucan, and the highly substituted forms of pectin (rhamnogalacturonan). Retention of enzymes that breakdown polygalacturonic acid and β-mannan, components of pollen tubes and flowers, respectively, illustrates the high degree of specialization of this fungus.

Whereas smut fungi generally cause necrosis as a space-making process during sporulation [85], *Microbotryum* anther smuts are more aptly characterized as a growth-altering parasite [86]. At the stage of anther development, *M. lychnidis-dioicae* causes abortion of pollen production, and replacement by the diploid teliospores for dispersal by pollinator species *[*87*].* Moreover, in the female, atrophy of pistils occurs during infection. There are a number of candidate pathways that might participate in pollen tube elongation or in blocking this process. Aspartyl proteases are involved in pollen tube elongation or prevention by *Metschnikowia reukaufii* [88]; seven candidate aspartyl proteases are predicted in *M. lychnidis-dioicae*. Additionally, consistent with transcriptional up-regulation of some component enzymes for the homogalactorunan degradation pathway of *M. lychnidis-dioicae*, this pathway may take over the role of pollen in anther dehiscence when the flowers bloom, since during teliospore formation of *M. lychnidis-dioicae*, host pollen is no longer available to perform that function Fig. 5.

Finally, since *M. lychnidis-dioicae* appears to lack a large repertoire for dealing with host-generated defenses that utilize reactive oxygen species (ROS) one could expect that the interaction with its host normally does not elicit such host responses or the fungus actively down-regulates them. Paradoxically, *M. lychnidis-dioicae* also appears to secrete some proteins that would serve to bring on this plant response by serving as prooxidants (*e.g.*, secreted gamma-glutamyltranspeptidases). Further elucidation of the roles of the predicted peroxidases superoxide dismutases, and glutathione-mediated detoxification pathway components will require functional analyses to evaluate their biological importance, if any, in the interaction of the pathogen with its hosts.

Secretory lipases represent one of the most significantly expanded gene families in *M. lychnidis-dioicae* compared to the other fungi examined. While haploid cells show no outward phenotype alteration in response to lipids like phytol, several secretory lipases of *M. lychnidis-dioicae* in haploid cells are up-regulated on nutrient limited agar. We propose that regulation of secretory lipases primes haploid cells so that, after mating, they can respond to the appropriate plant-derived cues (including lipids) to progress to the next developmental stage, stable dikaryotic hyphae. In fact, most of the secretory lipases we investigated were up-regulated in mated cells and when such cells were exposed to phytol (Additional file 18).

The identification of genes induced during infection (MI-late) suggests their involvement in host invasion and evasion of physical and chemical defense systems of the plant. A role for CAZymes, including pectin methylesterases and GHs, during plant infection in *M. lychnidis-dioicae* is further supported by increased transcription in the MI-late sample. Analysis of the gene expression profile of both wheat stem and poplar rust (*P. graminis* and *M. larici-populina*) also found that many CAZyme genes related to cell wall degradation were up-regulated during plant infection [39]. In addition to the CAZymes, *M. lychnidis-dioicae* shows significant induction of diverse transporters during plant infection, which may be critical for uptake of small molecules during biotrophic growth.

As in other plant pathogenic fungi, candidate effectors in *M. lychnidis-dioicae* were predicted based on predicted localization, expression during infection, and sequence conservation. Notably, SSPs are located closer to TEs than other protein coding genes, suggesting that this could impact SSP expression or duplication. TEs probably play a role in the expansion of such a family. Indeed they contribute to genome rearrangements and gene duplications [89]. In *Fusarium oxysporum* f. sp. *lycopersici,* effector genes are present on chromosomes or regions enriched for DNA transposons [90]. Some secreted proteins are predicted to act on host cell walls and proteins, either for the remodeling of host development in the flower or for acquisition of additional nutrients by the fungus.

In assembling sequence of the *a*_*1*_ mating-type chromosome, we characterized how the content of this allosome differs from autosomal regions, contrasting the non-recombining regions of the mating-type chromosome with the pseudo-autosomal regions (PARs) capable of recombination and with autosomes. Both the lower gene density and higher transposable element content in the non-recombining region of the mating-type chromosome relative to autosomal regions are consistent with a reduced efficiency of purifying selection due to the suppression of recombination, as occur on non-recombining sex chromosomes [59]. The two recombining PARs of the mating-type chromosome displayed TE content and gene density more similar to autosomes than the non-recombining part of the mating-type chromosome. By contrast, we observed no difference in codon usage nor in the GC content at third codon positions between autosomes and the mating-type chromosome, though this has been observed in the non-recombining part of the fungal mating-type chromosome of *Neurospora tetrasperma* [31]. Overall the maintenance of homologous meiotic pairing and recombination in PAR regions may render them more similar to autosomes than allosomes with respect to evolutionary forces of selection and drift. However, their physical linkage to the non-recombining region of mating-type chromosomes suggests intermediate modes of evolution [91].

## Conclusion

Altogether, this study provides an in-depth genomic portrait of a fungal castrating, biotrophic plant pathogen reflecting its unique life cycle. In particular, the unique absence of enzyme classes for plant cell wall degradation and maintenance of enzymes that break down components of pollen tubes and flowers provides a striking example of biotrophic host adaptation. In addition, while there are fewer enzymes to digest cellulose, xylan, xyloglucan, and highly substituted forms of pectin, as well as proteins that could protect the fungus from oxidative stress, the repertoire of predicted cell wall modifying enzymes and those that could manipulate host development has expanded (see model in Fig. 5). Given the place of *M. lychnidis-dioicae* in a large species-complex with a vast host species pool, the insights from this genomic and transcriptomic analysis combined with comparative approaches with other members of the *Microbotryum* species complex will be most informative on the evolutionary processes involved in a radiation and specialization on a wide array of plant species from different genera.

## Methods

### *Microbotryum lychnidis-dioicae* lineage(s) and *Silene latifolia* host(s)

The *focal* lineage of *M. lychnidis-dioicae* for this work is the most studied in the context of disease ecology (“Lamole strain”: GenBank I00-15Lamole.1; [9, 11]) and belongs to the recently-refined species designation *M. lychnidis-dioicae*, parasitizing *Silene latifolia*. From the original isolate, haploid sporidial strains were generated via micromanipulation of the meiotic products from a single tetrad, yielding the strains Lamole p1A1 and p1A2 that differ in electrophoretic karyotypes only in the mating-specific chromosome. For the work in this report, the haploid p1A1 strain (mating-type *a1*) was used as the source of the *focal* genome. Additionally, the *focal* genome contains size-heteromorphic sex chromosomes that share many features with sex chromosomes in plant and animal systems [11]. The corresponding *a*_*2*_ strain, p1A2 was used together with its partner strain p1A1 in plant infections and in RNA-Seq analysis.

### High molecular weight DNA preparation

*Microbotryum lychnidis-dioicae* Lamole p1A1 was grown on yeast peptone dextrose media (YPD; 1 % yeast extract, 10 % dextrose, 2 % peptone, 1.5 % agar) at room temperature for 5 days and ultimately extracted using a phenol chloroform isoamyl extraction method [92]. Harvested fungal cells were ground into fine powder using liquid nitrogen and resuspended in OmniPrep Genomic Lysis Buffer (G-Biosciences, cat no: 786–136) according to manufacturer’s recommended tissue to reagent ratio. The sample was heated in a 55-60 °C water bath for 15 min after extensive vortexing. Chloroform was added to the sample after allowing it to cool to room temperature. Using wide bore tips thereafter, 3–4 extractions using phenol chloroform isoamyl (25:24:1) solution were performed, followed by a final extraction with chloroform isoamyl (24:1) solution. Nucleic acid was then precipitated and the pellet was rinsed twice with ice-cold 70 % ethanol and then air-dried. The pellet was rehydrated using Tris-EDTA buffer (pH 8.0) (100 μl per 100 mg of ground tissue powder used) and treated with RNase (*Longlife* RNase, 5 mg/ml; G-Biosciences); 1 μl of RNase was added for every 100 μl of TE buffer used.

### RNA isolation

#### Haploid cells

Haploid fungal cells of either Lamole p1A1 or p1A2 grown separately under rich conditions for 5 days on yeast peptone dextrose media (YPD; 1 % yeast extract, 10 % dextrose, 2 % peptone, 2 % agar) at room temperature were harvested for RNA extraction. RNAs were checked for quality using a Bioanalyzer (Agilent). The RNAs were then pooled in equal quantity (in terms of mass) based on the Bioanalyzer quantification. The same procedure was also performed for the haploid cells grown separately on 2 % water agar for 2 days, to compare the gene expression when haploid cells were subjected to nutrient free environment without the mating partner. Again, haploid cell samples, p1A1 and p1A2, were extracted as independent samples, and then mixed in equal proportion for RNA sequencing.

#### Host plant infection for RNA-Seq (MI-late stage)

*Silene latifolia* seeds (harvested in Summer 2009 from Lamole, Italy) were sterilized and hydrated by soaking them in a sterilizing solution (40 % household bleach, 20 % absolute ethanol and 1 drop of Triton X-100 as surfactant per 50 ml of solution) and washing five times in sterile distilled water, for 2 min per wash with constant agitation. Each seed was then individually planted in closed milk jars on sterile 0.3 % phytagar (Life Technologies), 0.5× MS (Murashige and Skoog) salts (Sigma-Aldrich) and 0.05 % MES (2-(N-morpholino)ethanesulfonic acid) buffer (Brand). Each jar was placed at 4 °C for 5 days to synchronize germination. The jars were then transferred to a 20 °C growth chamber with 13 h of fluorescent light daily. Germination starts within 3 days with the appearance of the radicle. When the seedlings were 15 days old, they were transplanted into 2″ square pots filled with Sunshine MVP Professional Growing Mix (Sun Gro Horticulture Canada Ltd, cat no. 02392868) soil and replaced into the growth chamber. Humidity was kept high initially using dome covers and flood trays. Seedlings were gradually exposed to chamber environment for increasing amounts of time daily in order for the seedling to harden and adapt to the lower humidity. The plants were transplanted to 4″ round pots when they began to bolt at about 30 days old. They were further transplanted into 7″ round pots when they had almost attained maximum height or when the volume of soil was not sufficient to provide hydration requirement for the plant. The plants were watered every other day with 100-ppm fertilizer (Peters Professional® 15-16-17 Peat-Lite Special, Formula no: S12893).

To infect the host plants, mated cells were prepared as follows. Haploid cells grown on rich media were harvested and resuspended in distilled water, adjusted to a concentration of 1 × 10^9^ cells/ml in equal proportion before being spotted onto nutrient-free solid agar media (2 % agar) in 50 μl spots. The plates were allowed to dry and then incubated at 14 °C for about 48 h. Cells were inspected for conjugation tubes under the microscope and then 5 μl of 1× 10^6^ cells/ml resuspended in distilled water with anionic surfactant was pipetted onto the floral meristem when the cotyledon was fully developed (11–12 days). Infection was determined by the consistent blooming of fully smutted flowers. The floral buds were staged according to previous literature [35] under a dissecting scope (Nikon, Model: SMZ-U) and parts of the floral buds were measured with a glass stage micrometer (Imaging Research, Inc.).

Tissue originating from host plants was collected in RNAlater RNA stabilizing reagent (QIAGEN, cat no: 76106) and left at 4 °C overnight until sufficient tissue had been collected for the RNA extraction. The solution was removed before storing the sample at −80 °C. For infected male plants, we collected floral tissue from buds ranging in size from 4 mm to fully open smutted flowers. These tissue samples were pooled to yield the source for ‘MI-late’ RNA used in RNA-Seq analysis. Thus, they provide a pooled average picture of gene expression for this size range of infected tissue.

All RNA samples were extracted using the RNeasy Plant Mini Kit (QIAGEN, cat no: 74904) according to the manufacturer’s instructions. DNase treatment was performed using Ambion’s TURBO DNA-free (Applied Biosystems, cat no: AM1907), also according to manufacturer’s instruction. For quality assessment before Illumina sequencing, 5 μg of DNase-treated RNA was reverse transcribed with SuperScript III First Strand Synthesis System for RT-PCR (Life Technologies, cat no: 18080–051). PCR was performed using TaKaRa Ex Taq Hot-Start DNA Polymerase (Takara, cat. no: RR001B) using 25 μl reaction volume. To check for DNA contamination and possible inhibitory substances in the RNA, three sets of housekeeping primers (Eurofins/MWG/Operon) were used. Amplification of a region of the *S. latifolia* partial *wdr1x* gene for a putative WD-repeat protein (GenBank IDs Y18519, Aj310656) was used to assess host cDNA and contaminating genomic DNA; the forward primer, 5′- CTCTGCTGGAGGTGGAACAT-3′ and reverse primer, 5′- AGCACTGAACACCCCAACTT-3′; in this case a 253 bp fragment would be produced for cDNA, vs. a 335 bp fragment for genomic DNA. Targeting the *M. lychnidis-dioicae mepA* gene, we used as forward primer, 5′- CTTTTGCGTAGGAAGAATGC-3′ and as reverse primer, 5′- AGCACTGAACACCCCAACTT-3′; this combination yielded a 532 bp fragment from cDNA, compared with a 1039 bp fragment from genomic DNA. The other primer combination targeted the *M. lychnidis-dioicae* beta-tubulin gene, with forward primer, 5′- CGGACACCGTTGTCGAGCCT -3′, and reverse primer, 5′- TGAGGTCGCCGTGAGTCGGT-3′, yielding a 150 bp fragment from cDNA compared with a 215 bp fragment from genomic DNA. The PCR program was 30 s at 94 °C, 30 s at 60 °C and 1 min at 72 °C for 35 cycles. RNA quality was also evaluated using an Agilent BioAnalyzer; all samples had RNA integrity number scores of at least 7.8, indicating highly intact RNA.

### Treatment of cells with lipids

Haploid fungal cells of Lamole p1A1 and p1A2 were grown separately under rich conditions for 5 days on YPD at room temperature, then harvested into sterile distilled water. The concentration was adjusted and resuspended in equal proportions in each type of medium, to achieve a final concentration of 1 × 10^9^ cells/ml.

To allow better solubility of the lipids, 50 % ethanol was used as the solvent for the lipids. We used 1 % corn oil (Carlini), (±)-α-tocopherol (Sigma-Aldrich, cat no: T3251-5G), and phytol (Sigma-Aldrich, cat no: P3647), dissolved in the solvent and used as the resuspension media for the fungal cells. The mixtures were then spotted in 50 μl spots onto 2 % water agar and allowed to mate for 2 days at 14 °C. The cells were then observed under the microscope for conjugation tubes and filamentous structures. The solvent served as the control media to ensure that changes in phenotype were not due to the ethanol present.

### Genome and transcriptome sequencing, assembly, and annotation

For genome sequencing, we constructed three libraries (Additional file 1) with different insert sizes and sequenced each using 454 Technology. The reads were assembled with Newbler (version MapAsmResearch-04/19/2010-patch-08/17/2010). The total assembly size of 26.1 Mb in scaffolds includes 99.6 % of bases of at least Q40 quality; gaps encompass 3.45 % of the total scaffold length.

For RNA-Seq, we purified polyA RNA and constructed a strand-specific library for each sample as previously described [93, 94] and sequenced each with Illumina technology generating 76 base paired reads. Across the three libraries, 96 % of reads met the Illumina Passing Filter (PF) quality threshold. Read alignment rates to the genome varied between three libraries; for the rich and nutrient limited samples, 90 % or 89 % of reads aligned respectively; for the MI-late sample only 23 % of reads aligned. This was expected as these samples also contain the host *Silene* RNAs. To assemble transcripts for use in annotation, RNA-Seq reads were aligned to the assembly with Blat, and then assembled using Inchworm [95] in the genome-guided mode.

To predict genes, we first generated a high confidence training set of 775 transcripts of at least 900 nt using Genemark [96] and the assembled RNA-Seq data. This was used to train Augustus [97] and Glimmerhmm [98]. RNA-Seq data was processed by PASA [99] to generate longer transcripts, and ORFs of at least 600 nt were predicted. Available ESTs from Genbank and Microbase were also utilized. EVM [99] was then used to select a preliminary gene set from the *ab initio* gene calls (Augustus, Genemark, GlimmerHmm, and SNAP), Genewise [100], ESTs, PASA ORFs, and the training set, with the highest weight given to the RNA-Seq based PASA ORFs. The EVM gene set was compared to the PASA ORFs, and non-repeat genes found only in the PASA set were added to the gene set. Finally, PASA was run on the final gene set to all updates of all gene structures with the RNA-Seq and incorporate alternatively spliced transcripts. Genes likely corresponding to repeats were filtered out using TransposonPSI (requiring 1e-10 and 30 % overlap), PFAM domains, Blast similarity to repetitive elements and 7 or more Blast hits to other genes in the set. Genes with flagged features (proteins ≤50 aa, internal exons ≤6 nt, introns ≤20 nt, introns ≥1500 nt, exons spanning gaps in the assembly, internal stop codons, overlapping other coding sequences, overlapping ncRNAs (tRNAs, rRNA, or other)) were manually reviewed and corrected where supported by the evidence. Gene names were assigned with the locus prefix MVLG.

The completeness of the gene set was evaluated by examining the conservation and completeness of core eukaryotic genes (CEGs, [36]). We compared the gene set of *M. lychnidis-dioicae* and of the 18 other fungal genomes used in comparisons to the CEGMA set, and identified Blast hits above and below the recommended 70 % coverage threshold (Additional file 3). A tool for streamlined analysis and visualization of conservation of CEGs is available on SourceForge (http://sourceforge.net/projects/corealyze/).

### Differential expression analysis

We used differential expression analysis scripts in the Trinity pipeline [95, 101] to process RNA-Seq data generated from the three conditions (nutrient limited, rich, and MI-late). Briefly, we first extracted protein coding gene sequences from the *M. lychnidis-dioicae* genome sequence based on coordinates of gene models, and added 100 bases of flanking sequence on each side to approximate UTRs. Then the RNA-Seq reads from each of the three samples were aligned to the extracted coding sequences using bowtie [102]. The alignment files were used to quantify transcript abundances by RSEM [103]. Differential gene expression analysis was conducted using edgeR with TMM normalization [104, 105] using a corrected p-value [106] cutoff of 1e-3. In comparing all pairs of the three conditions, a total of 1,413 genes were differentially expressed across the comparisons (Additional file 24).

### TE detection and annotation

Two pipelines from REPET package (http://urgi.versailles.inra.fr/tools/REPET) were run on the *M. lychnidis-dioicae* contigs. The TEdenovo pipeline [107] was used to search for repeats in the genome. The first step uses Blaster with the following parameters [identity > 90 %, HSP (High Scoring segments Pairs) length >100b & <20Kb, *e*-value ≤ 1e-300]. HSPs found were clustered by 3 different methods: Piler [108], Grouper [109] and Recon [110]. Multiple alignments (MAP) of 20 longest members of each cluster (918 clusters) containing at least 3 members were used to derive a consensus. Consensus sequences were then classified based on their structure and similarities against Repbase Update (v15.11) [111] before removing redundancy (Blaster + Matcher). Consensus sequences without any known structure or similarity were classified as “Unknown”.

The library of 425 classified consensuses provided by the TEdenovo pipeline was used to annotate TE copies in the whole genome using TEannot pipeline [109]. Annotation is based on 3 methods (Blaster, Censor, RepeatMasker). HSPs provided were filtered and combined. Three methods (TRF, Mreps and RepeatMasker) were also used to annotate SSR. TE duplicates and SSR were then removed. Finally a “long join procedure” [107] was used to address the problem of nested TEs. This procedure finds and connects fragments of TEs interrupted by other TEs inserted more recently to build a TE copy. The nesting patterns of such insertion must respect the three constraints: fragments must be co-linear (both on the genome and the same TE consensus reference), have the same age and separated by younger TE insertion. The identity percentage with the reference consensus is used to estimate the age of a copy. Using results of this first TEannot pipeline, we filtered out 111 consensus sequences without full-length copy in the genome. A copy may be built using one or more fragments joined by the TEannot long join procedure. We ran a second TEannot using the 306 consensus elements remaining after filtering out TE consensus without any full-length copy.

We also used gene prediction and proceeded to manual curation in order to improve TE annotation. We removed TE copies of consensuses that were identified as host genes. Indeed, these consensuses built from family of repeats containing at least 3 members and classified as unknown by the TEdenovo pipeline has been predicted as host genes belonging to multigenic families. We also filtered out TE copies not satisfying the criteria (identity > 0.8 & length > 150 & identity*length > 150) and those corresponding to low complexity region of the consensus Mivi-B-R219-Map20_classI-LINE-incomp very highly represented in the genome included in predicted genes. The few copies just over these thresholds were manually removed, depending on their location in genes and evidence of the gene (PFAM domain not related to TEs).

### Search for signature of transition type (C to T) mutation bias

We performed pairwise alignments between each copy and respective consensus to finally provide multiple alignments for each family (consensus) using in-house scripts. TE copies with less than 80 % of identity with consensus and smaller than 400 bp were filtered out. We also filtered out TE families with less than 5 sequences in the multiple alignments. RIPCAL [112] was run on each multiple alignment to count both potential single mutations (transitions and transversions) and di-nucleotide target used in all possible transitions. Results were analysed using in-house R scripts to select most reliable mutated copies (if transition rate > 2 * transversion rate). For 40 % of copies exhibiting a transition mutation bias (of 2298 total copies, 179 consensus families (Additional file 10)), we considered that dinucleotide targets (CA + TG^1^, CC + GG^1^, CG + CG^1^, CT + AG^1^; ^1^ for reverse complement), were preferentially used if they represent a minimum of 30 % of the addition of the four possible. We expect 25 % of each if they are equiprobable.

### Measurement of distance between genes and TEs

We computed the distance from each gene to the closest TE (case 1), or from each TE to the closest gene (case 2) using getDistance.py from S-MART package [113]. Only distances up to 10 kb were considered. For case 1, we also compared the subset of genes encoding predicted secreted proteins with the set of all other genes for different classes of distance intervals. For the case 2, we compared different TE categories in two classes of distance intervals (<1kbp and >1kbp). A chi^2^ test of homogeneity (Pearson’s chi-squared) was computed to test that the observed difference between the sets did not occur by chance (p-value < 0.05). The graphics and statistical test were performed using in-house R-Scripts.

### Identification of the mating-type chromosome supercontigs

Using the same haploid genotype from which the whole genome was sequenced, DNA enriched for the *a*_*1*_ mating-type chromosome was isolated from agarose gels after pulsed-field electrophoresis. With this technique, the isolated bands could include of small amounts of autosomal fragments that co-migrate with mating-type chromosomes, though these preparations have been shown to be strongly enriched for mating-type chromosome DNA [12]. The isolated DNA was amplified by whole genome amplification (REPLI-g kit, QIAGEN). The DNA was sequenced using 2- and 5 kb-insert size mate-paired libraries and 454 technology version Titanium (www.roche.com). Assembly of non-duplicated reads and excluding autosomal contamination yielded ~20-fold coverage.

The assembly was compared to the *a*_*1*_ whole haploid genome sequence using NUCmer (http://mummer.sourceforge.net/) to validate assemblies and identify scaffolds corresponding to the mating-type chromosome in the whole genome assembly. The regions corresponding to autosomal contamination were identified by uneven and low read coverage and were excluded from further analyses; mitochondrial DNA was also excluded (GenBank NC_020353). All whole-genome scaffolds with more than 20-fold depth of the enriched mating-type chromosome sequence were confidently assigned to the mating-type chromosomes (Additional file 11). It was not possible to anchor the scaffolds onto the available optical map of the *a*_*1*_ mating-type chromosome [12] due to the small sizes of the contigs relative to spacing of the restriction enzyme cut sites in the map. Annotation for mapped supercontigs regions was parsed from the genome-level annotation.

TE content was assessed using de novo TE annotation as described above. TE content was compared between the nonrecombining part of the mating-type chromosome (Table 1), the PARs, and the autosomes. GC content was compared between the non-recombining region of the mating-type chromosome, the PARs and the autosomes. Significance of the mean difference was assessed using a *t*-test and a nonparametric Wilcoxon test. In a second step, the GC content at the 3rd codon positions was inspected separately in identified coding regions. Mean GC contents at the 3rd codon positions were compared between the CDS on the non-recombining part of the mating-type chromosome, the PARs and the autosomal coding regions. All GC content analyses were conducted using in-house python and R scripts.

### Prediction of the secretome

To predict a high confidence set of secreted proteins, results from several different software tools were integrated. These include TargetP1.1 [114], SignalP3.0, [115], SignalP4.0 (http://www.cbs.dtu.dk/services/SignalP/) [116], TMHMM2.0 [117], PredGPI [118], Phobius [119], NucPred [120], Prosite [121], and WoLF PSORT [122]. A subset of these tools were used to first exclude proteins as not secreted if they had transmembrane domains (two or more, from TMHMM or Phobius), an ER retention signal (0.00014 from Prosite), GPI anchor (specificity of >99.5 % using the general model of PredGPI), or nuclear localization (>0.8 threshold in NucPred). Secreted proteins were then predicted based on passing four of the six thresholds examined (TargetP secreted localization, SignalP3.0 NN Dscore > 0.43, SignalP3.0 HMM Sprob > 0.8, SignalP4.0 D-score > 0.45, WoLFPsort ‘Extr’ listed as major neighbor, or Phobius secreted localization).

Additional criteria were used for ambiguous predictions. If both TMHMM and Phobius agreed on the existence of 1–2 transmembrane (TM) domains in the protein, the protein was excluded from the probable secretome pool. If a protein was predicted to have a lowly probable GPI linkage (PredGPI specificity >99.0 % and <99.5 %) and a TM predicted by TMHMM and/or Phobius around the same region, this served as corroborating evidence for GPI anchorage to the membrane.

Where evidence conflicted or was insufficient for determining secretome status, BLASTp and Pfam domains were used to establish probable orthologs, followed by referencing the UniProtKB [123, 124] and FunSecKB [125] database for confirmation of localization of the orthologs, where available. Out of 7,360 predicted proteins, 6,899 proteins were excluded from the pool of the secretome based on the criteria described above. Of the remainder, 189 proteins had no contradictory calls in the positive prediction for SP. Another 272 went through further confirmation, of which 182 of these were confirmed to be non-SP and 63 were SP. Of the rest, 27 of them could not be finalized due to lack of ortholog matches in the NCBI database and lack of conserved domain for reference.

### Gene clustering and comparative analysis

We compared *M. lychnidis-dioicae* to 18 other fungi (Additional file 13) that sample the three subphyla in Basidiomycota, including 5 other Pucciniomycotina, 7 Agaricomycotina, 3 Ustilaginomycotina, as well as 3 Ascomycota outgroups. For *M. lychnidis-dioicae* and the 18 other fungal genomes, we identified ortholog clusters using OrthoMCL [126] version 1.4 with a Markov inflation index of 1.5 and a maximum e-value of 1 × 10^−5^. Two genomes, *R. glutinis* and *P. placenta*, are missing more broadly conserved orthologs than the other genomes; examining the 961 *Microbotryum* gene clusters with an ortholog missing in just one other genome, the number of missing clusters in any one Basidiomycete genome ranged from 1 to 34 with the exception of *R. glutinis* and *P. placenta*, missing 410 and 393 of these highly conserved clusters, respectively. PFAM domains within each gene were identified using Hmmer3 [127], and gene ontology terms were assigned using BLAST2GO [128].

To examine gene duplication history, the phylome, or complete collection of phylogenetic trees for each gene in a genome, was reconstructed for *Microbotryum lychnidis-dioicae* and 19 other fungi, including those used for OrthoMCL (Additional file 13) and *Serpula lacrymans*. Phylomes were reconstructed using the previously described pipeline [129]. All trees and alignments have been deposited in PhylomeDB [129] and can be browsed on-line (www.phylomedb.org, phylome code 180). Trees were scanned to detect and date duplication events [130].

RNAi components from other other fungi were used as Blast queries to find homologs in *M. lychnidis-dioicae*; the queries used include *U. hordei* RdRp (CCF48827.1), *C. neoformans* Ago1 (XP_003194007), and *N. crassa* Dcl2 (Q75CC1.3) and Dcl1 (Q758J7.1). The putative function was confirmed by examining protein domains. The identified domains for each protein include: Piwi, PAZ and DUF1785 found in both copies of Argonaute (MVLG_06823, MVLG_06899); DEAD/DEAH helicase, double-stranded RNA binding, and RNAseIII (MVLG_01202). Sugar transporters were identified based on homology to the *Ustillago maydis* Srt1t transporter (Genbank: XP_758521) and the *Uromyces viciae-fabae* Hxt1 (Genbank: CAC41332).

The *M. lychnidis-dioicae* protein models corresponding to carbohydrate-active enzymes were assigned to families of glycoside hydrolases (GH), polysaccharide lyases (PL), carbohydrate esterases (CE), carbohydrate-binding modules (CBM), auxiliary activities (AA) and glycosyltransferases (GT) listed by the CAZy database [64], exactly as previously done for the analyses of dozens of fungal genomes [39, 66, 131, 132].

### Data access

The assembly and annotation of *M. lychnidis-dioicae* was submitted to GenBank under accession number AEIJ01000000.

## Additional files

Additional file 1:
**is a table providing Genome sequencing statistics.**
Additional file 2:
**is a table showing RNA-Seq read statistics.**
Additional file 3:
**is a figure showing Conservation of core eukaryotic (CEGMA) genes.**
Additional file 4:
**is a figure Correlation between GC content and gene density.**
Additional file 5:
**is a figure presenting Preferred codons for the different amino-acids.**
Additional file 6:
**is a figure of Frequency of transposable element classes.**
Additional file 7:
**is a figure displaying Proximity of TE copies for main orders (Class I LTR and LINE, Class II TIR and Helitrons) to the closest gene.**
Additional file 8:
**is a figure presenting Comparison of TE proximity for secreted proteins compared to all other genes.**
Additional file 9:
**is a table that shows TE elements used in RIPCAL analysis.**
Additional file 10:
**is a figure that shows Frequency of mutation transition types in different TE classes.**
Additional file 11:
**is a table presenting Genes predicted on non-recombining mating type regions, pseudoautosomal regions (“PAR”) and autosomes.**
Additional file 12:
**is a figure presenting Codon usage in autosomal and mating-type-specific genome regions.**
Additional file 13:
**is a table showing Source of genome data used in comparative analysis.**
Additional file 14:
**is a table that shows Gene family duplications from Phylome analysis.**
Additional file 15:
**is a figure displaying Phylome for** ***M. lychnidis-dioicae.***
Additional file 16:
**is a figure of the Phylogenetic tree of DUF1034 proteins.**
Additional file 17:
**is a table with Species counts of enriched or depleted protein domains.**
Additional file 18:
**is a table that presents qRT-PCR validation of secretory lipase expression.**
Additional file 19:
**is a table with CAZymes content comparisons.**
Additional file 20:
**is a table that shows Phylogenetic comparison of CAZyme content.**
Additional file 21:
**is a figure showing growth of** ***M. lychnidis-dioicae*** **on different sole carbon sources, including dextrose, cellulose, xylan, and pectin [** 133]. Additional file 22:
**is a table of the Full set of predicted secreted proteins.**
Additional file 23:
**is a table showing Sequences similar to plant hormone-related genes.**
Additional file 24:
**is a table with Genes differentially expressed between nutrient limited, rich, and MI-late samples.**
Additional file 25:
**is a table of PFAM domains enriched in MI-late induced genes.**
