# Supplementary material for: Sex and parasites: genomic and transcriptomic analysis of Microbotryum lychnidis-dioicae, the biotrophic and plant-castrating anther smut fungus
Source: BMC Genomics. 2015 Jun 16;16(1):461. doi: 10.1186/s12864-015-1660-8 (PMC4469406; doi:10.1186/s12864-015-1660-8)
Supplement: Additional file 1: — is a table providing Genome sequencing statistics. [file 12864_2015_1660_MOESM1_ESM.docx]

**Additional file 1. Genome sequencing statistics.**

| 454 Library Insert | Sequencing Type | Molecular Barcode | Reads (count) | Average Read Length (bases) | PF Bases (count) | Sequence Depth (Fold) |
| --- | --- | --- | --- | --- | --- | --- |
| Fragment | Single read | Madrid | 1,145,609 | 371.3 | 425,393,315 | 16.36 |
| 2.7 kb | Paired read | Bratislava | 510,499 | 280.2 | 143,013,765 | 5.50 |
| 5.5 kb | Paired read | Cairo | 686,469 | 162.2 | 111,354,758 | 4.28 |
| 5.5 kb | Paired read | Budapest | 233,405 | 189.1 | 44,129,203 | 1.70 |
| Total |  |  | 2,575,982 |  | 723,891,041 | 27.84 |
