# Supplementary material for: Sex and parasites: genomic and transcriptomic analysis of Microbotryum lychnidis-dioicae, the biotrophic and plant-castrating anther smut fungus
Source: BMC Genomics. 2015 Jun 16;16(1):461. doi: 10.1186/s12864-015-1660-8 (PMC4469406; doi:10.1186/s12864-015-1660-8)
Supplement: Additional file 2: — is a table showing RNA-Seq read statistics. [file 12864_2015_1660_MOESM2_ESM.docx]

**Additional file 2. RNA-Seq read statistics.**

| Sample | Reads | PF* Reads | PF Reads aligned** | PF Reads aligned (%)** |
| --- | --- | --- | --- | --- |
| Haploid rich | 17,025,508 | 16,239,792 | 14,613,444 | 89.99% |
| Haploid nutrient limited | 19,275,930 | 18,335,352 | 16,366,386 | 89.26% |
| MI-late | 20,165,846 | 19,575,594 | 4,441,268 | 22.69% |
| Total | 56,467,284 | 54,150,738 | 35,421,098 |  |

*Illumina Passing Filter high quality criteria.

**Number and percent of PF reads aligned to *M. lychnidis-dioicae* genome
