# Supplementary material for: Sex and parasites: genomic and transcriptomic analysis of Microbotryum lychnidis-dioicae, the biotrophic and plant-castrating anther smut fungus
Source: BMC Genomics. 2015 Jun 16;16(1):461. doi: 10.1186/s12864-015-1660-8 (PMC4469406; doi:10.1186/s12864-015-1660-8)
Supplement: Additional file 3: — is a figure showing Conservation of core eukaryotic (CEGMA) genes. [file 12864_2015_1660_MOESM3_ESM.docx]

**Additional File 3.** **Conservation of core eukaryotic genes (CEGs) set across *M. lychnidis-dioicae* and other fungal genomes.** The percent coverage of genes with significant Blast similarity is shown for alignments above and below the recommended 70% coverage threshold, which can indicate partial gene structures.
