# Supplementary material for: Sex and parasites: genomic and transcriptomic analysis of Microbotryum lychnidis-dioicae, the biotrophic and plant-castrating anther smut fungus
Source: BMC Genomics. 2015 Jun 16;16(1):461. doi: 10.1186/s12864-015-1660-8 (PMC4469406; doi:10.1186/s12864-015-1660-8)
Supplement: Additional file 4: — is a figure Correlation between GC content and gene density. [file 12864_2015_1660_MOESM4_ESM.docx]

**Additional file 4. Correlation between GC content and gene density.**

A . Using the assembly generated from 454 sequencing, GC percent was computed in non-overlapping windows of 10 kbp and plotted against genomics coordinates. Vertical blue lines represent the limits between scaffolds. B. Gene density was also measured in the same windows as in A. There was a significant positive correlation (p-value < 2.3e-05) between density in (CDS) and GC percent, explaining 16.8 % of the variance.
