# Supplementary material for: Sex and parasites: genomic and transcriptomic analysis of Microbotryum lychnidis-dioicae, the biotrophic and plant-castrating anther smut fungus
Source: BMC Genomics. 2015 Jun 16;16(1):461. doi: 10.1186/s12864-015-1660-8 (PMC4469406; doi:10.1186/s12864-015-1660-8)
Supplement: Additional file 5: — is a figure presenting Preferred codons for the different amino-acids. [file 12864_2015_1660_MOESM5_ESM.docx]

**Additional file 5. Preferred codons for each amino-acid.** The frequencies of the different codons are shown for each amino-acids. The most frequent codons often show higher GC content.
