# Supplementary material for: Sex and parasites: genomic and transcriptomic analysis of Microbotryum lychnidis-dioicae, the biotrophic and plant-castrating anther smut fungus
Source: BMC Genomics. 2015 Jun 16;16(1):461. doi: 10.1186/s12864-015-1660-8 (PMC4469406; doi:10.1186/s12864-015-1660-8)

**Additional file 6**. **Distribution of transposable elements in *M. lychnidis-dioicae* genome according to their TE classification.** A. Genome coverage (%) of TEs according to their order; B. TE space coverage (%) of TEs according to superfamilies of the major orders (LTR, LINE, DIRS retro-transposons, TIR, Helitron and MITE DNA transposons).

A B


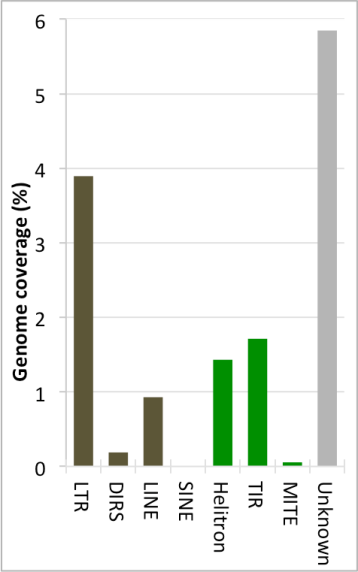

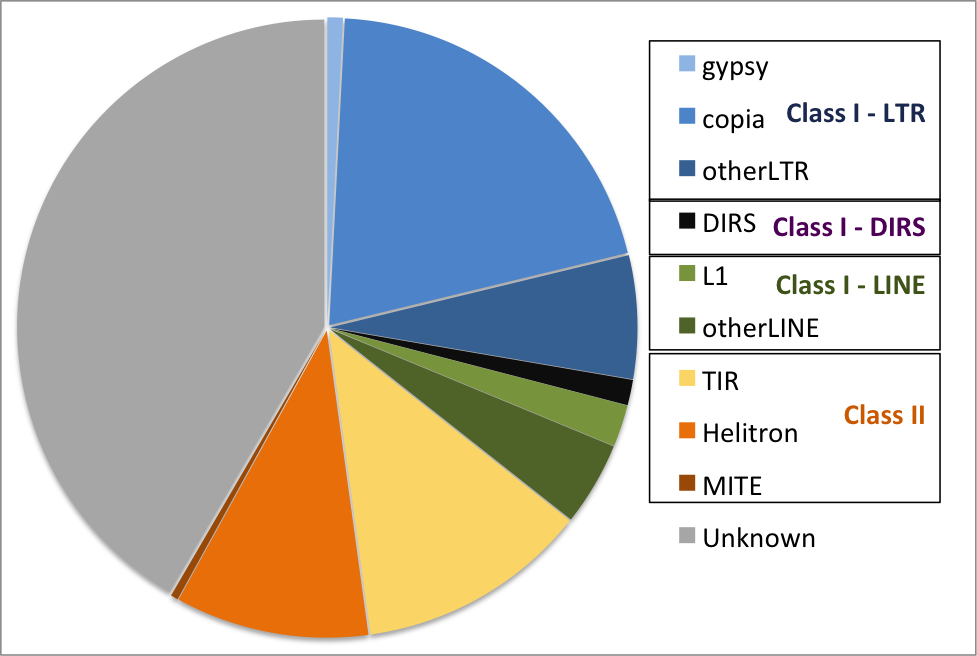

Supplement: Additional file 6: — is a figure of Frequency of transposable element classes. [file 12864_2015_1660_MOESM6_ESM.docx]
