# Supplementary material for: Sex and parasites: genomic and transcriptomic analysis of Microbotryum lychnidis-dioicae, the biotrophic and plant-castrating anther smut fungus
Source: BMC Genomics. 2015 Jun 16;16(1):461. doi: 10.1186/s12864-015-1660-8 (PMC4469406; doi:10.1186/s12864-015-1660-8)

**Additional file 7. Proximity of TE copies for main orders (Class I LTR and LINE, Class II TIR and Helitrons) to the closest gene.** Two classes of distance were compared: < 1kb and > 1kb. A. Upstream regions of genes (Pearson's Chi-squared test p-value < 2.2e-16) and B. Downstream regions of genes (Pearson's Chi-squared test p-value < 2.2e-08)


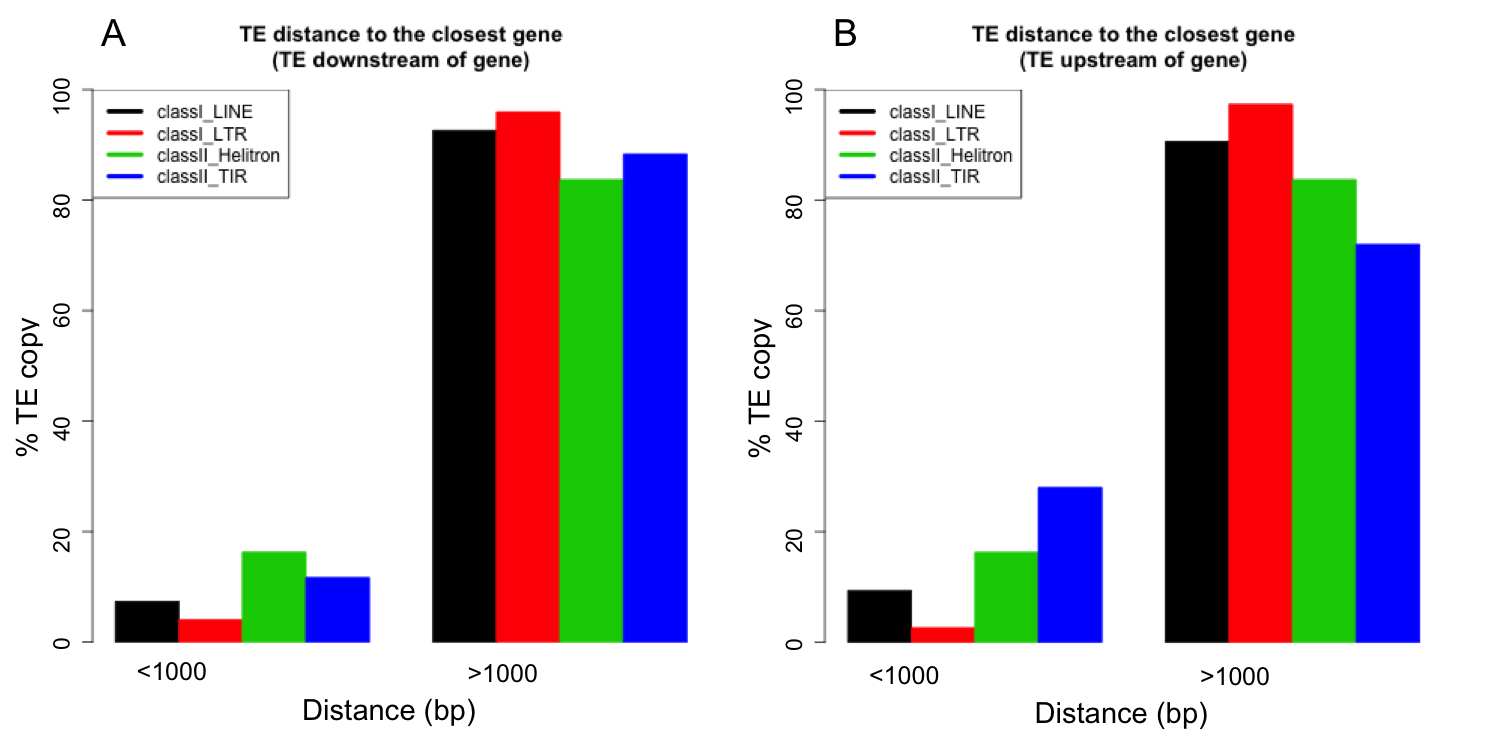

Supplement: Additional file 7: — is a figure displaying Proximity of TE copies for main orders (Class I LTR and LINE, Class II TIR and Helitrons) to the closest gene. [file 12864_2015_1660_MOESM7_ESM.docx]
