# Supplementary material for: Sex and parasites: genomic and transcriptomic analysis of Microbotryum lychnidis-dioicae, the biotrophic and plant-castrating anther smut fungus
Source: BMC Genomics. 2015 Jun 16;16(1):461. doi: 10.1186/s12864-015-1660-8 (PMC4469406; doi:10.1186/s12864-015-1660-8)

**Additional file 8. Comparison of TE proximity for secreted proteins compared to all other genes.** The percent of genes (Gene %) is shown for either A. Two classes of distance 0-1kb and >1kb (Pearson's Chi-squared test p-value = 5e-4) and B. Ten classes of distance from 0-1 kb, to 9-10 kb (Pearson's Chi-squared test p-value = 1e-2).

A B


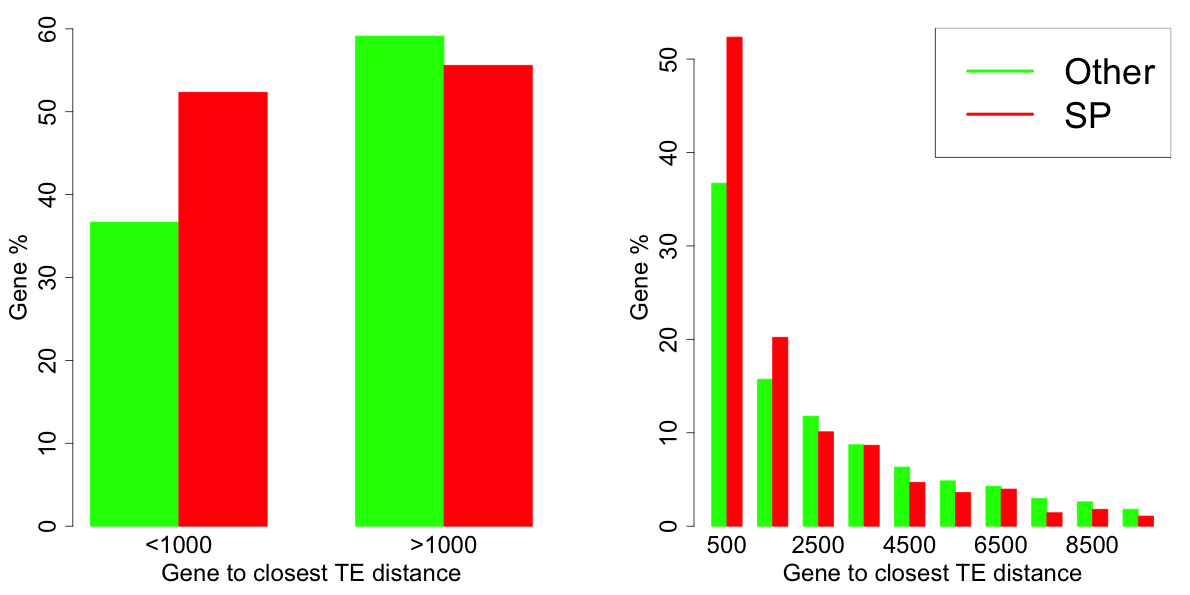

Supplement: Additional file 8: — is a figure presenting Comparison of TE proximity for secreted proteins compared to all other genes. [file 12864_2015_1660_MOESM8_ESM.docx]
