# Supplementary material for: Sex and parasites: genomic and transcriptomic analysis of Microbotryum lychnidis-dioicae, the biotrophic and plant-castrating anther smut fungus
Source: BMC Genomics. 2015 Jun 16;16(1):461. doi: 10.1186/s12864-015-1660-8 (PMC4469406; doi:10.1186/s12864-015-1660-8)
Supplement: Additional file 9: — is a table that shows TE elements used in RIPCAL analysis. [file 12864_2015_1660_MOESM9_ESM.docx]

**Additional file 9. TE used in RIPCAL analysis.** 2,298 copies from 179 TE families were used in RIPCAL calculations. GC% is calculated on whole sequences used.

| LTR families | LTR copies | | DIRS  families | DIRS copies | | LINE families | | LINE copies | TIR families | | TIR copies | | MITE  families | MITE  copies | Helitron  families | Helitron copies | Unknown  families | Unknown copies |
| --- | --- | --- | --- | --- | --- | --- | --- | --- | --- | --- | --- | --- | --- | --- | --- | --- | --- | --- |
| 37 | 569 | | 2 | 24 | | 11 | | 203 | 18 | | 300 | | 1 | 10 | 15 | 264 | 95 | 928 |
| GC%  copies | | GC%  genes | | | GC%  genome | |  | | |  | |  |  |  |  |  |  |  |
| 54.4 | | 56.04 | | | 55.43 | |  | | |  | |  |  |  |  |  |  |  |
