# Supplementary material for: Sex and parasites: genomic and transcriptomic analysis of Microbotryum lychnidis-dioicae, the biotrophic and plant-castrating anther smut fungus
Source: BMC Genomics. 2015 Jun 16;16(1):461. doi: 10.1186/s12864-015-1660-8 (PMC4469406; doi:10.1186/s12864-015-1660-8)

**Additional file 10. Frequency of mutation transition types in different TE classes.** For each TE type, the frequency of di-nucleotide substitutions are plotted; these are measured between each copy (total of 2,298 copies) and the highest GC content sequence in a multiple alignment of the TE consensus and the corresponding genome copies. Colored bars: percentage of copies with expected RIP-like mutation (if Transition rate > 2 * Transversion rate) and DI-nucleotide preferentially used >30% in CN->TN and (cNG -> cNA) mutations. Black bar: percentage of copies without expected RIP-like (Transition rate < 2 * Transversion rate). Note that the CA+CG+CC+CT percentage of copy could be over than 100% if copies exhibit more than one bias.


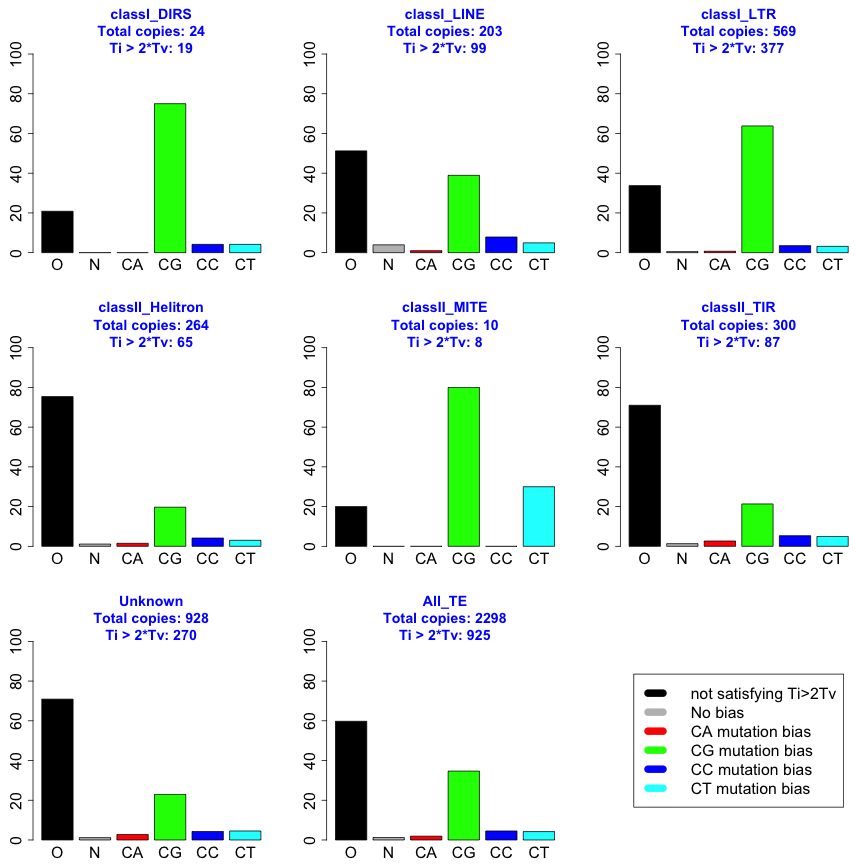

Supplement: Additional file 10: — is a figure that shows Frequency of mutation transition types in different TE classes. [file 12864_2015_1660_MOESM10_ESM.docx]
