# Supplementary material for: Sex and parasites: genomic and transcriptomic analysis of Microbotryum lychnidis-dioicae, the biotrophic and plant-castrating anther smut fungus
Source: BMC Genomics. 2015 Jun 16;16(1):461. doi: 10.1186/s12864-015-1660-8 (PMC4469406; doi:10.1186/s12864-015-1660-8)
Supplement: Additional file 12: — is a figure presenting Codon usage in autosomal and mating-type-specific genome regions. [file 12864_2015_1660_MOESM12_ESM.docx]

**Additional file 12. Codon adaptation of autosomal and sex-specific genome regions.** Correspondence analysis of synonymous codon usage, which compares sets of genes belonging to non-recombining mating type regions, pseudoautosomal regions (“PAR”) and autosomes.
