# Supplementary material for: Sex and parasites: genomic and transcriptomic analysis of Microbotryum lychnidis-dioicae, the biotrophic and plant-castrating anther smut fungus
Source: BMC Genomics. 2015 Jun 16;16(1):461. doi: 10.1186/s12864-015-1660-8 (PMC4469406; doi:10.1186/s12864-015-1660-8)
Supplement: Additional file 15: — is a figure displaying Phylome for M. lychnidis-dioicae. [file 12864_2015_1660_MOESM15_ESM.docx]

**Additional file 15. Phylome for *Microbotryum lychnidis-dioicae.*** Maximum likelihood tree of 20 fungal species including *M. lychnidis-dioicae*, and other basidiomycete and ascomycete species. The tree has been rooted by the midpoint method for clarity. The alignments of 52 proteins with a single ortholog in each of the 20 studied species were concatenated into a single trimmed alignment  of 48,538 positions. The phylome in this study was reconstructed using the pipeline described in (Huerta-Cepas et al. 2011) and can be browsed on-line ([www.phylomedb.org](http://www.phylomedb.org/), phylome code 180).
