# Supplementary material for: Sex and parasites: genomic and transcriptomic analysis of Microbotryum lychnidis-dioicae, the biotrophic and plant-castrating anther smut fungus
Source: BMC Genomics. 2015 Jun 16;16(1):461. doi: 10.1186/s12864-015-1660-8 (PMC4469406; doi:10.1186/s12864-015-1660-8)
Supplement: Additional file 17: — is a table with Species counts of enriched or depleted protein domains. [file 12864_2015_1660_MOESM17_ESM.docx]

**Additional file 17. Phylogenetic tree of DUF1034 proteins.** Protein sequences were aligned with MUSCLE, and a phylogeny was inferred from this alignment using RAxML with the following settings: substitution model: PROTCAT; Matrix name: DAYHOFF; algorithm: (d) Hill-climbing-default and *N. crassa* as the outgroup. A total of 1,000 bootstrap replicates were performed, with the option Bootstrap random seed (b) 12345, and the percent of replicates shown on the tree nodes. All *M. lychnidis-dioicae*  representatives are indicated by the MVLG_ prefix; other prefixes correspond to other species as follows: NCU, *N. crassa*; Srei, *S. reilianum*; UM, *U. maydis*; CNA, *C. neoformans*; CC1G, *C. cinereus*; Pchr, *P. chrysosporium*; LBIC, *L. bicolor*; PIIN, *P. indica*; PGTG, *P. graminis*; Mlp, *M.* *larici-populina*.
