# Supplementary material for: Sex and parasites: genomic and transcriptomic analysis of Microbotryum lychnidis-dioicae, the biotrophic and plant-castrating anther smut fungus
Source: BMC Genomics. 2015 Jun 16;16(1):461. doi: 10.1186/s12864-015-1660-8 (PMC4469406; doi:10.1186/s12864-015-1660-8)
Supplement: Additional file 18: — is a table that presents qRT-PCR validation of secretory lipase expression. [file 12864_2015_1660_MOESM18_ESM.docx]

**Additional file 18. qRT-PCR validation of secretory lipase expression**

| Strain(s) | Condition | Gene | Log fold Change vs. p1A1 Rich | Direction |
| --- | --- | --- | --- | --- |
| p1A1 | Water | MVLG_07291 | 0.77 | Up |
|  |  | MLVG_00914 | 0.327 | Up |
|  |  | MLVG_05549 | 1.922 | Up |
|  |  | MLVG_07229 & 07284^a^ | 1.899 | Up |
|  |  |  |  |  |
| p1A2 | Water | MVLG_07291 | 1.84 | Up |
|  |  | MLVG_00914 | -1.308 | Down |
|  |  | MLVG_05549 | 0.327 | Up |
|  |  | MLVG_07229 & 07284 | 2.034 | Up |
|  |  |  |  |  |
| Mated (p1A1 x p1A2) | Water | MVLG_07291 | 1.424 | Up |
|  |  | MLVG_00914 | -1.098 | Down |
|  |  | MLVG_05549 | 0.672 | Up |
|  |  | MLVG_07229 & 07284 | 1.828 | Up |
|  |  |  |  |  |
| Mated (p1A1 x p1A2) | Phytol | MVLG_07291 | 0.913 | Up |
|  |  | MLVG_00914 | 1.2 | Up |
|  |  | MLVG_05549 | 1.499 | Up |
|  |  | MLVG_07229 & 07284 | 2.098 | Up |
|  |  |  |  |  |
| Mated (p1A1 x p1A2) | MI-late | MVLG_07291 | -1.897 | Down |
|  |  | MLVG_00914 | -1.772 | Down |
|  |  | MLVG_05549 | 0.77 | Up |
|  |  | MLVG_07229 & 07284 | -0.564 | Down |

^a^The primers used could not distinguish between MVLG_07229 & 07284; so results are presented here for both, assuming their expression is similar.
