# Supplementary figures and images for: Sex and parasites: genomic and transcriptomic analysis of Microbotryum lychnidis-dioicae, the biotrophic and plant-castrating anther smut fungus
Source: BMC Genomics. 2015 Jun 16;16(1):461. doi: 10.1186/s12864-015-1660-8 (PMC4469406; doi:10.1186/s12864-015-1660-8)

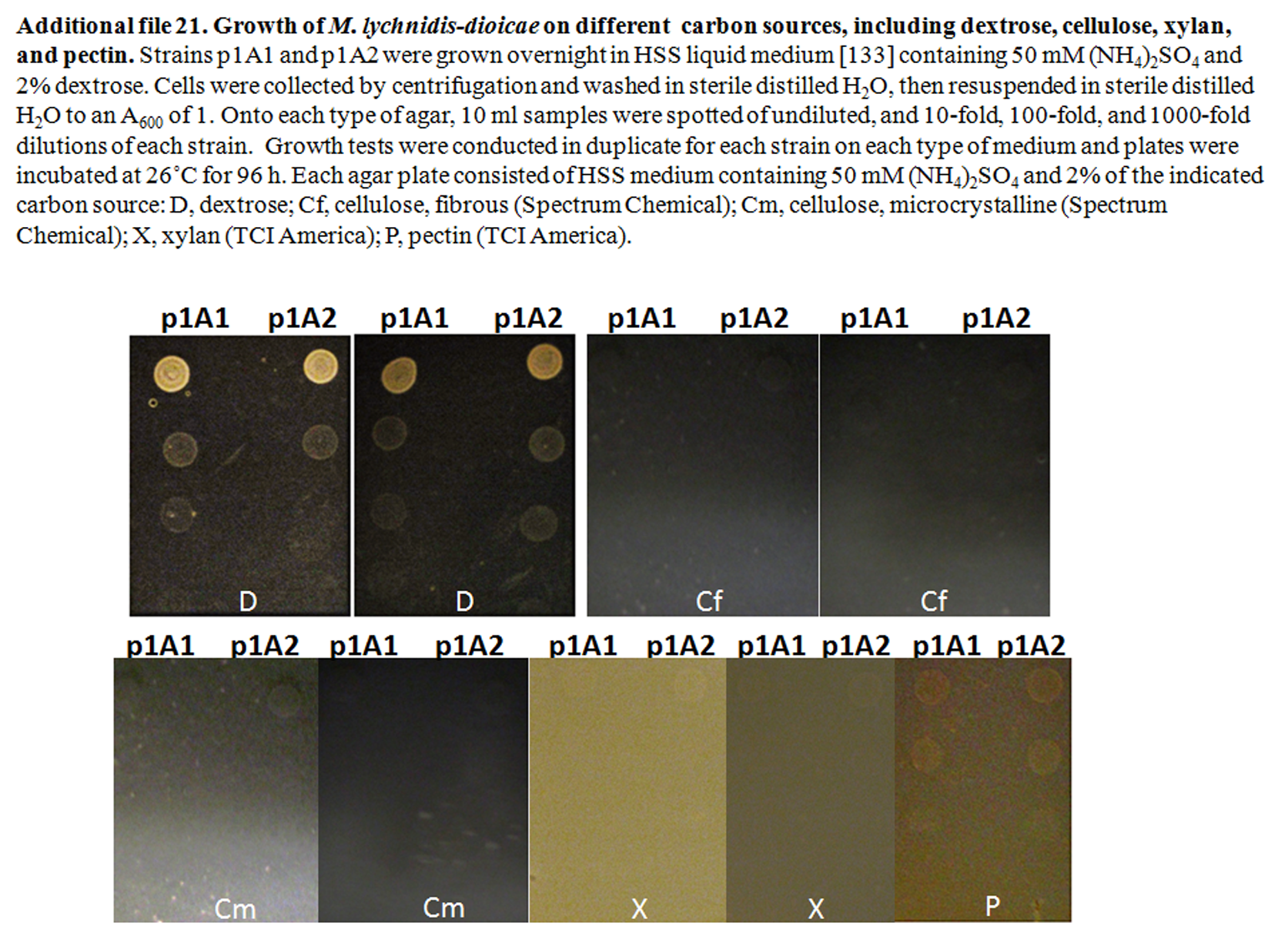

Supplement: Additional file 21: — is a figure showing growth of M. lychnidis-dioicae on different sole carbon sources, including dextrose, cellulose, xylan, and pectin [ 133]. [file 12864_2015_1660_MOESM21_ESM.tiff]
