# Supplementary material for: Sex and parasites: genomic and transcriptomic analysis of Microbotryum lychnidis-dioicae, the biotrophic and plant-castrating anther smut fungus
Source: BMC Genomics. 2015 Jun 16;16(1):461. doi: 10.1186/s12864-015-1660-8 (PMC4469406; doi:10.1186/s12864-015-1660-8)
Supplement: Additional file 25: — is a table of PFAM domains enriched in MI-late induced genes. [file 12864_2015_1660_MOESM25_ESM.docx]

**Additional file 25. PFAM domains enriched in MI-late induced genes.**

| PFAM domain | MI-late Induced genes | Other genes | Fisher pvalue | Corrected pvalue |
| --- | --- | --- | --- | --- |
| PF07690.9 Major Facilitator Superfamily | 15 | 113 | 8.42E-07 | 0.0011 |
| TIGR00879 MFS transporter, sugar porter (SP) family | 6 | 14 | 7.96E-06 | 0.0051 |
| PF07250.4 Glyoxal oxidase N-terminus | 4 | 4 | 2.83E-05 | 0.0091 |
| PF09118.4 Domain of unknown function (DUF1929) | 4 | 4 | 2.83E-05 | 0.0091 |
| PF00295.10 Glycosyl hydrolases family 28 | 3 | 3 | 0.0003 | 0.0838 |
| PF00128.17 Alpha amylase, catalytic domain | 3 | 4 | 0.0006 | 0.0962 |
| PF01590.19 GAF domain | 3 | 4 | 0.0006 | 0.0962 |
| PF03169.8 OPT oligopeptide transporter protein | 4 | 12 | 0.0006 | 0.0962 |
| PF05978.9 Ion channel regulatory protein UNC-93 | 2 | 0 | 0.0007 | 0.0962 |
